# Interleukin-1 Gene Cluster Polymorphisms and Their Association with Coronary Artery Disease: Separate Evidences from the Largest Case-Control Study amongst North Indians and an Updated Meta-Analysis

**DOI:** 10.1371/journal.pone.0153480

**Published:** 2016-04-14

**Authors:** Himanshu Rai, Nakul Sinha, Sudeep Kumar, Ajay Kumar Sharma, Suraksha Agrawal

**Affiliations:** 1 Department of Cardiology, Sanjay Gandhi Post Graduate Institute of Medical Sciences, Lucknow, Uttar Pradesh, India; 2 Department of Zoology, University of Lucknow, Lucknow, Uttar Pradesh, India; 3 Department of Medical Genetics, Sanjay Gandhi Post Graduate Institute of Medical Sciences, Lucknow, Uttar Pradesh, India; CSIR-INSTITUTE OF GENOMICS AND INTEGRATIVE BIOLOGY, INDIA

## Abstract

Several researchers have reported significant association of numerous single nucleotide polymorphisms (SNPs) residing in the interleukin-1 (IL-1) gene cluster with coronary artery disease (CAD). However, their association status amongst North Indian ancestry (NIA) have never been systematically assessed. Despite a published meta-analysis on this subject, their association status worldwide as well as amongst different major ancestral subgroups still remains unclear. We therefore decided to prospectively test the association of 11 IL-1 gene cluster SNPs with CAD, vide a case-control study amongst a cohort of NIA and attempted to validate our results with the help of an updated meta-analysis of all relevant published association studies. Included studies were segregated into ancestral subgroups and association statuses for each subgroup were determined. A total of 323 cases and 400 healthy, age and sex matched controls belonging to NIA were prospectively enrolled and subsequently genotyped for 11 selected IL-1 gene cluster SNPs. Although results for none of the evaluated IL-1 gene cluster SNPs reached the adjusted level of significance (p<0.0045), clear trends of association were seen for *IL1B* -511 C>T and *IL1RN* 86bp VNTR in several of the constructed genetic models (p range = 0.01–0.044 and 0.005–0.034 respectively). The presence of >1, ‘T’ (minor) allele of *IL1B* -511 C>T in a genotype seemed to provide protection against CAD (OR = 0.62, p = 0.044), while the presence of >1, ‘C’ (major) allele seemed to increase the risk of CAD (OR = 1.36, p = 0.041). The minor allele (allele 2) of *IL1RN* 86bp VNTR and its homozygous genotype (2/2 genotype) also seemed to carry an increased risk for CAD (OR = 1.62, p = 0.005 and OR = 2.25, p = 0.031 respectively). On the other hand, several haplotype combinations constructed out of *IL1B* and *IL1RN* gene variants clearly showed statistically significant associations with CAD (p<0.0045). Our meta-analysis was conducted for 8 previously assessed IL-1 SNPs. We included 53 different studies which involved a total sample of 26,210 (13,982 cases and 12,228 controls). Our pooled results concurred with the findings of our case-control study and was not able to deduce any statistically significant associations for any of the 8 studied SNPs (p>0.05). Subgroup analysis, however, yielded interesting results, where significant differences in association statuses were seen for *IL1A* +4845 G>T, *IL1B* -511 C>T, *IL1RN* 86bp VNTR and *IL1RN* +8006 T>C for select ancestral subgroups. The hints of associations deduced for subjects belonging to NIA in our case-control study for both *IL1B* -511 C>T and *IL1RN* 86bp VNTR were duly validated vide significant p values seen for NIA in all three genetic models (OR range = 0.62–0.76, p range = 0.01–0.04 and OR range = 1.51–2.25, p range = 0.004–0.04 respectively). On the other hand, Mixed Ancestry (MA) subgroup carrying *IL1B* -511 C>T, *IL1RN* 86bp VNTR or *IL1RN* +8006 T>C polymorphisms seemed to enjoy significant protection against CAD. A few other ancestral subgroups also demonstrated significant associations for a few of the studied SNPs vide one of the three genetic models. Clinical interpretation of derived results is however recommended.

## Introduction

Coronary artery disease (CAD) in many ways is an inflammatory disease. Inflammation plays an important role in the formation of atheroma which ultimately graduates to atheromatous injury, plaque rupture and coronary thrombosis precipitating into a major cardiovascular event.[1] Presence of increased levels of inflammatory markers in ruptured atheromatous plaques validates the association between inflammation and CAD.[2] Cytokines belonging to Interleukin-1 (IL-1) family has been known to be a critical and early mediator in various immunoinflammatory mechanisms.[3] IL-1 family cytokines have previously been implicated in the regulation of endothelial and smooth muscle cell mitogenesis, thrombogenic response of endothelial cells, leukocyte adherence, lipoprotein metabolism, extracellular matrix production and vascular permeability.[4, 5] IL-1 family is also known to be involved specifically in the process of plaque formation and rupture via different pathways: (a) by stimulation of vascular smooth muscle cells vide transforming growth factor-β (TGF-β),[6] (b) suppression of endothelial cell proliferation,[7] (c) expression of adhesion molecules by endothelial cells,[8] and (d) by modification of endothelium which later favours thrombosis.[9] Interleukin-1 has two agonists (IL-1α and IL-1β) and one antagonist (IL-1Ra), which are encoded respectively by *IL1A*, *IL1B* and *IL1RN* genes. These three genes are encompassed in the interleukin-1 gene cluster which is located within a 430 kb region on chromosome 2 (2q13-21). All these three genes are highly polymorphic entailing several single nucleotide polymorphisms (SNPs); some of them among which have even been implicated with CAD. Over the years, the associations reported have been area specific and sporadic. A meta-analysis of association studies published on the subject in 2012, reported lack of association of 3 SNPs (i.e. *IL1B* -511 C>T, *IL1B*+3954 C>T and *IL1RN* 86bp VNTR) residing in IL-1 gene cluster with CAD.[10] However, since risk of a genetically heritable disease is known to differ in different ethnic populations, we hypothesized that investigating the association of IL-1 gene cluster SNPs with CAD within several specific ancestral groups employing an updated meta-analysis of association studies might end up generating valuable information on the subject. Apart from a single published case-control study among North Indians (which examined only 1 SNP i.e. *IL1A*-889 C>T from this gene cluster), [11] no other association study examining IL-1 gene cluster SNPs for their role in CAD have been conducted among the population belonging North Indian ancestry. Therefore, a relatively large, prospective, case-control study, devised in order to test the association of known IL-1 gene cluster polymorphisms with CAD amongst North Indians was warranted.

Taking the above facts into consideration, our primary aim was to test the association of various known polymorphisms of the IL-1 gene cluster, namely: *IL1A*-889 C>T (rs1800587), *IL1A*+4845 G>T (rs17561), *IL1B*-1903 C>T (rs1143627), *IL1B*-3954 C>T (rs1143634), *IL1B*-5887 C>T(rs1143633), *IL1B*-511 C>T (rs16944), *IL1RN*+9589 A>T (rs454078), *IL1RN*+8006 T>C (rs419598), *IL1RN*+8061 C>T (rs423904), *IL1RN*+111000 T>C (rs315952) and *IL1RN* 86bp VNTR (PMID 14563376) and their role in the pathogenesis of CAD amongst subjects belonging to North Indian ancestry. Our secondary aim was to validate our results, and to assess the association statuses of selected IL-1 gene cluster polymorphisms with CAD. For this purpose, we planned to conduct an updated meta-analysis clubbing our study results with the results of all relevant association studies on the subject published so far. In addition to the pooled analysis, we also planned to conduct a subgroup analysis in order to assess the association statuses of these SNPs separately amongst different ancestral subgroups.

## Materials and Methods

### Inclusion and exclusion criteria

In the present study, a total of 323 proven CAD patients and 400 age and sex matched controls belonging to North Indian ancestry were prospectively included from 8 secondary/tertiary care teaching hospitals located in Uttar Pradesh (UP), which is the most populated province situated in the northern part of India. The present study was the genetic sub-study of UPCSI (Uttar Pradesh Cardiological Society of India)-Lipid study,[12] which was a unique, prospective, multicentric study among patients belonging to North Indian ancestry. Cases were included in the present genetic sub-study, if they satisfied at least one of the three inclusion criteria listed below: (a) History of MI (chest pain with ECG changes and/or elevated cardiac enzymes); (b) Angiographically proven CAD (>50% stenosis in at least one major epicardial artery); (c) History of stable or unstable angina along with positive Treadmill Test (TMT) or ST-T changes on Electrocardiogram (ECG) with positive cardio-specific enzymes (either Creatine-kinase (CK)-MB or Troponin T/I). Patients with debilitating disease, Pregnant females, lactating mothers, and elderly subjects (>75 years) were excluded from the study. Controls for the study were also sampled during the same time period, from the aforementioned study centers. Controls were healthy volunteers (hospital staff and attendants of patients with unrelated conditions) and were free from any of the conventional risk factors i.e. premature family history of CAD, hypertension and diabetes. Institutional ethics committees of all the participating centers prospectively approved the study protocol, subject questionnaire and standardized consent forms. The eight institutional ethics committees (IECs) belonging to eight participating centers in Uttar Pradesh which approved the study and various study materials were: (1) IEC, Sanjay Gandhi Post Graduate Institute of Medical Sciences (SGPGIMS), Lucknow; (2) IEC, King George’s Medical University (KGMU), Lucknow; (3) IEC, Ganesh Shankar Vidyarathi Medical College (GSVM), Kanpur; (4) IEC, Moti Lal Nehru (MLN) Medical College, Allahabad; (5) Ethical Committee of faculty of Medicine, Institute of Medical Sciences, Banaras Hindu University (BHU), Varanasi; (6) Medicity Ethical Committee, Varanasi; (7) IEC, Maharani Laxmibai Medical (MLM) College, Jhansi and (8) IEC, Baba Raghav Das (BRD) Medical College, Gorakhpur. Written consent was obtained from each subject before inclusion which was followed by data collection on a uniform questionnaire. Five ml of fasting whole blood in Ethylenediaminetetraacetic acid (EDTA) along with 5 ml of serum (isolated from plain blood) was collected at inclusion for genetic and biochemical analysis respectively. Collected whole blood and serum samples along with their corresponding filled questionnaires were then shipped to the coordinating centre in Lucknow, UP, India for analysis. All laboratory analysis (biochemical as well as genetic) for all included subjects was performed in a central facility in order to ensure standardization.

### Biochemical, genetic and statistical analyses

Protocols employed for biochemical analysis are explained in detail in our previous publication,[12] whereas genetic analysis for selected IL-1 gene cluster polymorphisms was done using polymerase chain reaction-restriction fragment length polymorphism (PCR-RFLP) method. Deoxyribonucleic acid (DNA) was isolated from the already collected EDTA whole blood using standard phenol chloroform method and was later used for genotyping using techniques adopted from previously published reports for various selected *IL1A*,[13] *IL1B*[13, 14] and *IL1RN*[14, 15] SNPs. Genotyping for *IL1RN* 86bp VNTR was performed using PCR followed by standard 2% agrose gel electrophoresis employing already published methodology.[15]

Genotypic frequencies for selected IL-1 gene cluster polymorphisms were determined by direct gene counting method, and the differences in the observed genotype/allele frequencies between case and control cohorts were tested by appropriate statistical test (Pearson’s Chi square or Fisher’s exact test). Departure from Hardy-Weinberg equilibrium amongst both case and controls, for each studied genetic variant was assessed by goodness-of-fit *x*^*2*^ test, assuming a p<0.05 as a cutoff value. The haplotype combinations in patient and controls (separately) were determined by using Computer program POPGEN VER. 1.32 (http://www.ualberta.ca/~fyeh/fyeh). Haplotype frequencies amongst cases and controls were then compared using a windows based statistical program called “GRAPHPAD”. All the baseline data was computed using SPSS 16.0. Data quality assurance and exploratory data analysis was performed before final analysis. Pearson’s Chi square test (χ^2^), Fisher’s exact test and student’s t-test were used as applicable. All hypothesis testing was done assuming a two tailed test. Since 11, IL-1 gene cluster variants were chosen to be tested in the present case-control study, Bonferroni’s correction yielded p value of 0.0045 was used as our threshold of significance, rather than a traditional p value of 0.05.

### Methods used for meta-analysis

We adhered to the Preferred Reporting Items for Systematic Reviews and Meta-Analyses-PRISMA statement [16] and the specific recommendations for genetic meta-analysis in the HuGE Review Handbook, version 1.0 while undertaking this project.

#### A. Search strategy and data collection

The databases of the US National Institutes of Health (PubMed), EMBASE, MEDLINE, Scopus and Web of Knowledge were systematically searched. Relevant articles published online till 30^th^ Jun 2015 were considered. Search headings as well as open text fields were used to identify papers. Databases and the reference lists of the relevant publications were searched using the combination of terms like ‘Interleukin 1’ OR ‘IL1’ OR ‘Interleukins’ paired with ‘coronary artery disease’ OR ‘CAD’ OR ‘Myocardial Infarction’ OR ‘MI’ OR ‘Acute Myocardial Infarction’ OR ‘AMI’ OR ‘Acute Coronary Syndrome’ OR ‘ACS’ AND ‘polymorphism’ OR ‘mutation’ OR ‘Single Nucleotide Polymorphism’ OR ‘SNP’. The search was restricted to articles relating to humans, covering all relevant English language publications. The decision to include studies was hierarchical; initially study titles, then abstracts and finally the full body of the text were assessed. To be included in the meta-analysis, articles had to assess the association between CAD or MI patients and CAD free controls. Our selection criteria included studies that met all of the following criteria: (1) published in a peer-reviewed journal and independent studies using original data; (2) unrelated case-control studies and cohort studies; (3) providing complete data with genotype and allele frequencies to calculate the odds ratio (OR) with confidence interval (CI) and p values; (4) CAD patient diagnosis based on coronary angiography/clinical assessment and controls not being CAD patients; (5) all studies included had to be published in English language; (6) Genotype frequency among control population for all included IL-1 variants should satisfy Hardy-Weinberg equilibrium (HWE). Departure from HWE amongst controls was checked by goodness-of-fit *x*^*2*^ test. Studies with control populations not conforming to HWE approximations, i.e. those with resultant p <0.05, were excluded. Abstracts published in conference brochures/abstract books, case reports, case studies and studies not providing adequate information on selection criteria and the actual distribution of polymorphisms in each group were excluded. Among the total of 11 selected IL-1 gene cluster SNPs, quantitative synthesis was only conducted, if we found at least 1 additional published study through our comprehensive literature search. All publications which lacked enough data, in order to generate all three genetic models were identified and formal requests for required data were made to their corresponding authors via periodic emails. We later included the publications among which the data available was enough to construct at least one genetic model. The publications in which no relevant data was made available even after three consecutive email requests to their corresponding authors (spaced one week apart) were finally excluded. The list of totally and partially excluded studies excluded from the present meta-analysis is updated in S1 File. The raw data was recorded from the relevant studies, on a paper proforma and was then transcribed on a MS-Excel worksheet, where further calculations (if needed) were done. Apart from the pooled calculations, subgroups were created for each SNP, categorizing studies based on the major ancestry studied. The created subgroups were labeled as, European Ancestry (EA), Middle Eastern Ancestry (MEA), Asian Ancestry (AA), North Indian Ancestry (NIA), African Ancestry (AFA) and Mixed Ancestry (MA). It is well known that environmental factors play a crucial role in determining the association status of a genotype, especially for multifactorial diseases/conditions like CAD. However, algorithms of complex statistical techniques such as meta-analyses are not designed to determine the impact of this complex relationship. Also since all the data required for determining the effect of environmental factors is not freely available and researchers are expected to work only with the available data, therefore, an adjustment of environmental factors in included individual studies in the present meta-analysis was not possible and thus not attempted.

#### B. Quality Assessment

The quality of included studies was systematically assessed using the Newcastle-Ottawa scale for non-randomized studies (http://www.ohri.ca/programs/clinical_epidemiology/oxford.asp), which is a star rating system.[17] This scale defines separate procedures for evaluating both case-control as well as cohort studies. In the aforementioned rating system, a study with a full score fetches 9 stars. A score between 5 to 9 stars indicates a good quality study, while a score of 0–4 stars indicates a poor quality study.[18, 19] The quality of studies in this scale was assessed primarily by (a) evaluating selection methods of cases and controls (or study cohort), (b) assessment of comparability status amongst cases and controls (or study cohort), and (c) ascertainment of exposure/outcome amongst case and controls (or study cohort). Quality assessment for all included studies was done independently by two authors and any disagreement was resolved through deliberation.

#### C. Statistical techniques

All calculations in the present meta-analysis were carried out using Review Manager (*RevMan*) [Computer program]. Version 5.3. Copenhagen: The Nordic Cochrane Centre, The Cochrane Collaboration, 2012. The extracted data from all publications were tested using dominant, recessive and allelic genetic models.

Bivariate and random or fixed effect models were used for calculating odds ratios (OR’s). For each genetic variant, individual and summary odds ratios and their 95% confidence intervals (CIs) were calculated for all genetic models, using either random (DerSimonian-Laird method)[20] or fixed effects model (Mantel-Haenszel method).[21] The analytic model was selected as per the observed heterogeneity within the studied group. The calculated OR for each study with its corresponding 95% CI was used to reveal the nature of association. Based on the individual ORs, a pooled OR was estimated, the significance of which was determined by *Z* test (p <0.05 showed statistical significance, and the corresponding Z value showcased the level of association).

Existence of heterogeneity was tested using a Q test. Tests for heterogeneity were performed using Higgins *I*^*2*^ statistics (*I*^*2*^) and Cochran’s Q statistics (P_Q_) for each analysis and the study group with a P_Q_ cut-off value of <0.1 was identified as a heterogeneous group. Low, moderate and high heterogeneity was defined according to previously published estimates[22] using cut off points of *I*^*2*^ values of 25%, 50% and 75% respectively. Criterion for selecting fixed effects for analysis in a group/subgroup was predefined with a cut-off P_Q_ value of 0.10. An attained P_Q_ value of >0.10 in a group/subgroup qualified it for the use of fixed affects for analysis, whereas random effects were used for analysis of groups/subgroups with P_Q_ value of ≤0.10. Subgroup differences were assessed assuming the same cut-off P_Q_ value. Differences yielding a P_Q_ value of ≤0.10 were assumed to be significant, while others (P_Q_> 0.10) were assumed to be non-significant.

Since most of statistical methods available for detection of publication bias are sensitive to heterogeneity, we used two of the most accepted methods in our updated meta-analysis. Presence of publication bias among the studied groups/subgroups (with ≥3 included studies) was visually detected by the use of Begg’s funnel plots[23] and its estimates were calculated by Egger’s test.[24]. Begg’s funnel plot was constructed for pooled analysis of each SNP and for each genetic model (with ≥3 included studies), while Egger’s estimates in the form of a p value was calculated for pooled as well as its major ancestral groups (having ≥3 included studies). An Egger’s p value of <0.05 was considered as statistically significant, which indicated possibility of publication bias within the studied group.

Sensitivity analysis was performed in each study group in every genetic model, where we excluded studies one after another and conducted the analysis after each omission. We tested if the results in any of the groups and studied genetic models altered substantially to change the results from non-association to significant association or the other way around (in groups of ≥ 5 studies). Absence of such phenomenon generally indicates the robustness of that meta-analysis.

## Results

The baseline characteristics of the case and control groups are showcased in S1 Table. The controls were non-diabetics, non-hypertensives, had no premature family history of CAD. The cases and control groups in our study were matched for age, sex and ethnicity. The case group had significantly higher percentage of smokers as compared to controls (43.34% vs. 31.75% respectively, p = 0.001). The mean levels of various lipid sub-fractions (except for very low density lipoprotein cholesterol: VLDL), their derived ratios along and fasting glucose levels were also found to be significantly higher among cases than in controls (p≤ 0.001). Presentation diagnoses of the subjects included in our case cohort is also duly presented in S1 Table.

Genotype frequencies for all studied SNPs, both among cases and controls satisfied Hardy-Weinberg approximations. Since we tested 11 variants in our present case-control study, a Bonferroni’s correction was applied. Our adjusted threshold of significance (i.e. p-value) came out to be as 0.0045. Although, none of the SNPs belonging to IL-1 family, technically provided statistically significant evidence of association (p>0.0045), a couple of them i.e. *IL1B* -511 C>T and *IL1RN* 86bp VNTR certainly showed trends of association with the disease. (S2 Table) The T (minor) allele and the TT genotype for *IL1B* -511 C>T polymorphism showed a promising trend towards protective nature against CAD (OR = 0.76, p = 0.013 and OR = 0.62, p = 0.044 respectively). Comparisons in co-dominant, dominant and recessive genetic models also did not reach the adjusted statistical significance, but hinted towards the protective nature of the resultant T allele and the TT genotype. The C (major) allele and CC genotype of *IL1B* -511 C>T thus automatically hinted to be involved with an increased CAD risk. On the other hand, minor allele 2 (240 bp) and genotype 2/2 resulting from *IL1RN* 86bp VNTR polymorphism showed a trend to be associated with increased risk for CAD (OR = 1.62, p = 0.005 and OR = 2.25, p = 0.031 respectively). This effect was further inspected, when we performed comparisons after renaming all other alleles (than allele 2) as allele X. Their genotypes were thus renamed as X/X, X/2 and 2/2. Results from comparisons (using the previously described nomenclature) among dominant, recessive and allelic models, although did not reach adjusted statistical significance (p> 0.0045), but showed similar trends of association of genotype 2/2 and allele 2 with CAD. (S2 Table)

Haplotype analysis was performed separately for *IL1A*, *IL1B* and *IL1RN* SNPs. Several haplotype combinations for SNPs belonging to *IL1B* and *IL1RN* genes showed significant association with CAD (p< 0.0045). Several such haplotypes either imparted additional risk or proved to be a protective factor against CAD. None of the constructed haplotype combinations of *IL1A* gene variants however showed any hint of association with the disease. (S3 Table)

### Meta-analysis results

**Fig 1:**
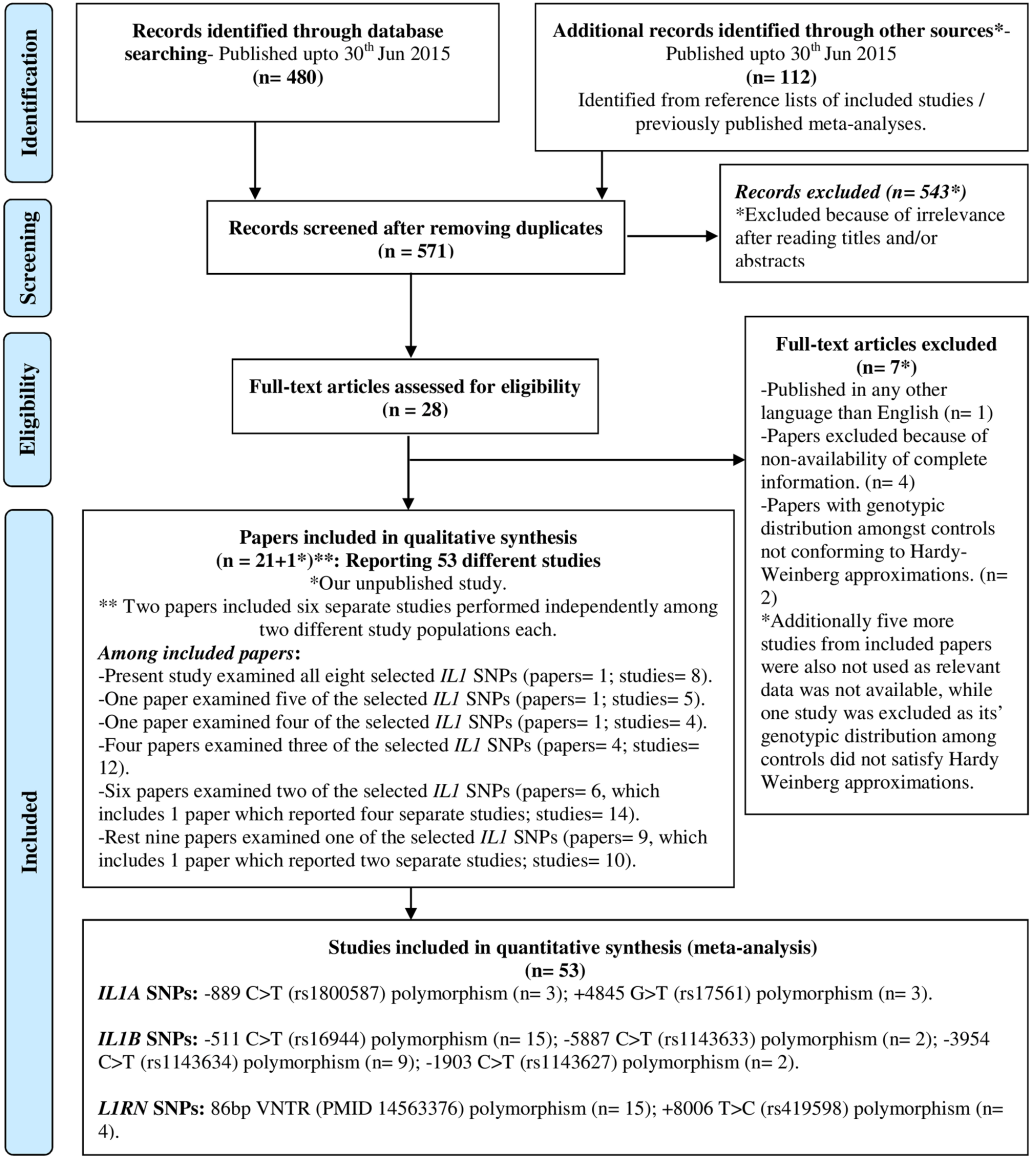
Meta-Analysis: Study selection flowchart.

In an effort to find relevant studies to be included in the present meta-analysis, we initially identified a total of 571 published papers through a comprehensive internet database search. Among them a total of 543 irrelevant articles were excluded after examination of their titles and/or abstracts (e.g. case studies, reviews, meta-analyses or with non-relevant outcome definitions etc.). This left us full texts of 28 articles to be assessed for eligibility. Among them 7 papers were excluded for a variety of reasons e.g.: non-availability of complete information (n = 4), genetic distribution amongst controls not conforming to Hardy-Weinberg approximations (n = 2) and published in any other language than English (n = 1). Finally 21 papers (plus 1 present article), with 53 different studies (including 8 from the present manuscript) were included for quantitative synthesis. Since we were not able to find a single relevant and qualifying, published study for 3 *IL1RN* gene SNPs, viz. +111000 T>C, +8061 C>T and +9589 A>T, they were excluded from quantitative synthesis and the data for the rest 8 SNPs was meta-analyzed. (Fig 1) Overall data from 53 different studies (21 articles + 1 present article), with a total sample of 26,210 (13,982 cases and 12,228 controls) was analyzed in the present meta-analysis. (S4 Table) All included studies were good quality studies which fetched at least 6 out of 9 stars when evaluated using the Newcastle-Ottawa scale.[17] Information about each study included in quantitative synthesis in each ancestral subgroup, like their countries of origin, studied outcome, sample size, reported minor allele frequencies for each studied SNP and their Newcastle-Ottawa rating[17] is given in Table 1.

**Table 1:** Association studies included in the present meta-analysis.

Study	Year	Predominant ancestry	Country	IL-1 gene cluster SNPs studied	MAF (Cases/Controls)	Total sample size (Cases/Controls)	Outcome	Newcastle-Ottawa scale rating
Francis et al.(a)-(Sheffield)[28]	1999	European	UK	B-511, RN VNTR	0.32/0.26 and 0.26/0.23 respectively	555 (425/130) for each	CAD	8/9 stars
Francis et al.(b)- (London)[28]	1999	European	UK	B-511, RN VNTR	0.32/0.32 and 0.28/0.16 respectively	350 (248/102) for each	CAD	8/9 stars
Iacoviello et al.[39]	2000	European	Italy	RN VNTR	0.25/0.25	311 (158/153)	PMI	8/9 stars
Momiyama et al.[37]	2001	Asian	Japan	B-511	NA/NA	292 (188/104)	CAD	6/9 stars
Zee et al.[40]*	2001	European	USA	RN VNTR	0.25/0.26	770 (385/385)	MI	9/9 stars
Vohnout et al.[29]	2003	European	Slovakia	B-511, RN VNTR	0.32/0.34 and 0.23/0.26 respectively	540 (335/205) for each	CAD	6/9 stars
Licastro et al.[30]	2004	European	Italy	B-511	0.32/0.36	261 (139/122)	MI	7/9 stars
Iacoviello et al.[31]	2005	European	Italy	B-511, B+3954	0.30/0.36 and 0.21/0.20	825 (406/419) for B-511; 800 (398/402) for B-3954	MI	7/9 stars
Kariz et al.[41]	2007	European	Slovenia	RN VNTR	0.26/0.23	374 (151/223)	MI	7/9 stars
Arman et al.[34]	2008	Middle Eastern	Turkey	B-511, B+3954, RN VNTR	0.45/0.48, 0.23/0.25 and 0.25/0.22 respectively	427 (257/170) for each	CAD	7/9 stars
Geismar et al.[32]	2008	European	Denmark	B-511, B+3954, RN VNTR	0.33/0.31, 0.27/0.27 and 0.21/0.29 respectively	219 (96/123) for B- 511, and B-3954; 217 (95/122) for RN VNTR	CAD	8/9 stars
Soylu et al.[36]	2008	Middle Eastern	Turkey	B-511, B+3954, RN VNTR	0.45/0.51, 0.23/0.23 and 0.25/0.30 respectively	381 (264/117) for each	ACS	7/9 stars
Zee et al. [26]*	2008	European	USA	A+4845, B-511, B-5887, B+3954, RN+8006	0.32/0.32, 0.34/0.32, 0.33/0.35, 0.26/0.24 and 0.25/0.25 respectively	682 (341/341) for each	MI	9/9 stars
Banerjee et al.[11]	2009	North Indian	India	A-889	0.32/0.29	442 (210/232)	CAD	8/9 stars
van Minkelen et al.[44]	2009	European	Netherlands	RN+8006	0.28/0.24	1205 (559/646)	MI	9/9 stars
Stein et al.[25]	2009	European	Germany	A-889, B+3954	NA/NA for both	141 (72/69) for each	AMI	9/9 stars
Fragoso et al.[42]	2010	Mixed	Mexico	RN VNTR, RN+8006	0.26/0.35 and 0.25/0.32 respectively	548 (300/248) for VNTR; 537 (289/248) for RN+8006	ACS	7/9 stars
Rios et al.(a)-(African-Brazilians)[33]	2010	African	Brazil	B-511	0.46/0.54	253 (138/115)	CAD	8/9 stars
Rios et al.(b)-(Caucasian-Brazilians)[33]	2010	European	Brazil	B-511	0.47/0.41	414 (276/138)	CAD	8/9 stars
Coker et al.[35]	2011	Middle Eastern	Turkey	B-511, B+3954, RN VNTR	0.43/0.43, 0.28/0.24 and 0.32/0.31 respectively	402 (167/235) for each	MI	9/9 stars
Goracy et al.[38]	2011	European	Poland	B-1903, RN VNTR	0.37/0.36 and 0.32/0.31 respectively	318 (201/117) for each	CAD	8/9 stars
Rywik et al.[43]	2011	European	Poland	RN VNTR	0.21/0.31	186 (110/76)	CAD	7/9 stars
Bashour et al.[27]	2013	Middle Eastern	Syria	A-4845, B-511, B+3954, RN VNTR	NA/NA for all	200 (100/100) for each	CAD	7/9 stars
Present study	2014	North Indian	India	A-889, A-4845, B-511, B-5887, B+3954, B-1903, RN VNTR, RN+8006	0.09/0.08, 0.10/0.10, 0.32/0.38, 0.26/0.26, 0.19/0.19, 0.37/0.39, 0.13/0.09 and 0.07/0.06 respectively	723 (323/400) for each	CAD	8/9 stars

IL-1: Interleukin-1; SNPs: Single nucleotide polymorphisms; MAF: Minor allele frequency; CAD: Coronary artery disease; MI: Myocardial infarction; ACS: Acute coronary syndrome; PMI: Premature MI

Interleukin-1 SNPs included- *IL1A*-889 C>T (rs1800587); *IL1A*+4845 G>T (rs17561); *IL1B* -511 C>T (rs16944); *IL1B* -5887 C>T (rs1143633); *IL1B* -3954 C>T (rs1143634); *IL1B* -1903 C>T (rs1143627); *IL1RN* 86bp VNTR (PMID 14563376); *IL1RN* +8006 T>C (rs419598).

* Cohort studies (Rest were case-control studies).

**Fig 2:**
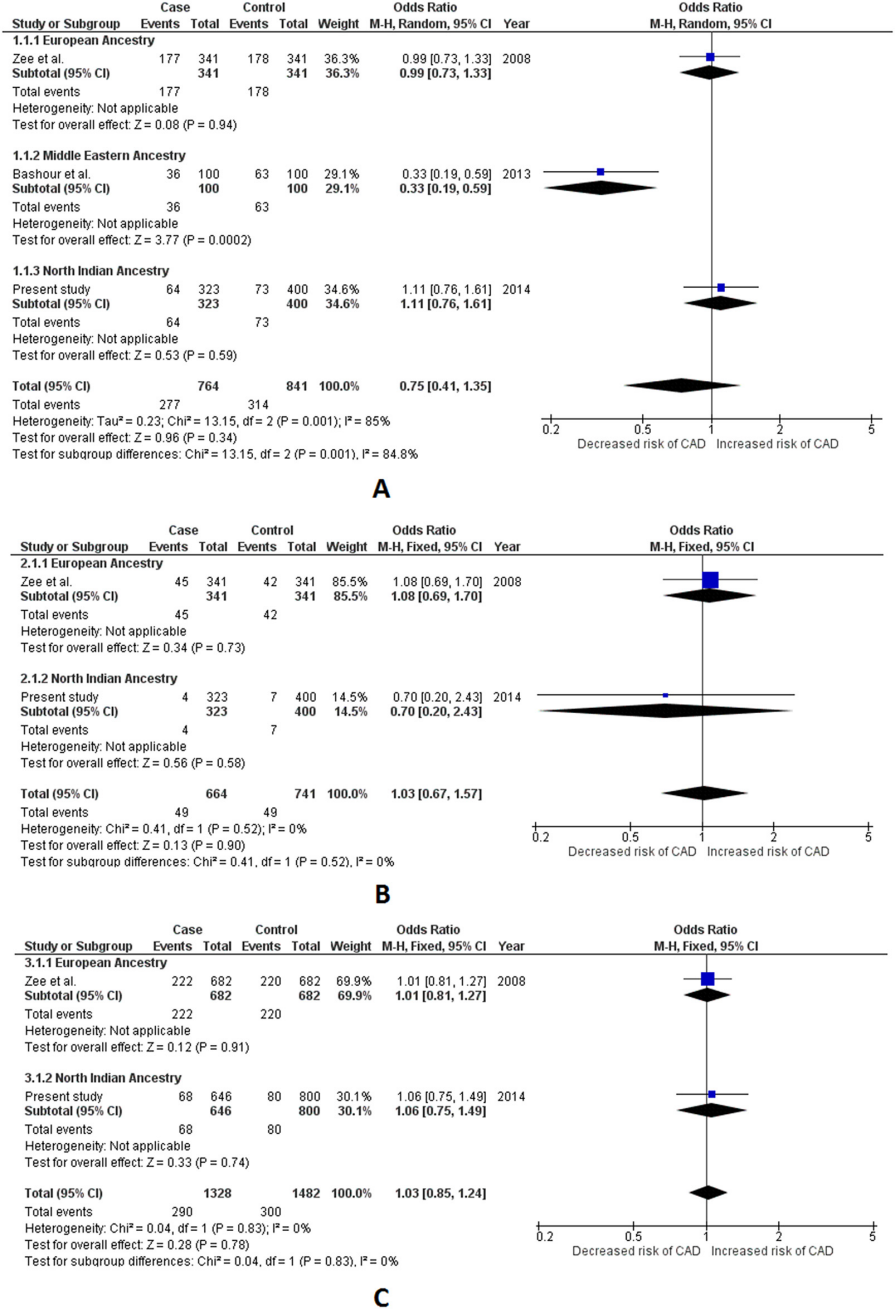
Forest plots depicting meta-analysis results for *IL1A* +4845 G>T (rs17561) polymorphism. Panel A: Effect size estimation using dominant genetic model (*TT+GT vs*. *GG*); Panel B: Effect size estimation using recessive genetic model (*TT vs*. *CT+GG*); Panel C: Effect size estimation using allelic genetic model (*Allele T vs*. *Allele G*). Pooled effect size estimates for dominant genetic model in Panel A was obtained using random effects for analysis, while fixed effects were used for effect size estimation for all ancestral groups. Revised effect size estimates for all ancestral groups analyzed in dominant genetic model are given in Table 2. Fixed effects were used for effect size estimation in recessive and allelic genetic models for pooled as well as all ancestral groups.

Two variants of *IL1A* gene viz. -889 C>T and +4845 G>T were selected for meta-analysis. We included a total of three studies, including ours’,[11, 25] to be used for quantitative synthesis for -889 C>T with a data of 1,306 subjects (605 cases and 701 controls). Appropriate effects for pooled analysis were used for each genetic model comparisons based on its inherent heterogeneity. Lack of association with CAD for -889 C>T was however indicated by our pooled results as obtained in each of the studied genetic models (p value range = 0.27–0.57). No significant association with CAD for -889 C>T was detected either, for EA and NIA in our subgroup analysis vide all the three genetic models (p >0.05). There was also no evidence of existing subgroup differences (*I*^*2*^< 50% and P_Q_> 0.10). (Table 2 and S4 Table, S1 Fig) We found only 3 studies (including ours’) fit for inclusion for +4845 G>T [26, 27] with a total sample size of 1,605 subjects (764 cases and 841 controls). Pooled results obtained using appropriate effects for quantitative synthesis suggested lack of association with CAD in all of the studied genetic models (p value range = 0.34–0.90). Apart from the MEA, in which +4845 G>T polymorphism (in the dominant genetic model) demonstrated to be significantly associated with lower odds for CAD (OR = 0.33, p = 0.0002), no association was seen in either of the remaining ancestral subgroups. Significant subgroup differences were observed in the dominant genetic model (*I*^*2*^ = 84.8%, P_Q_ = 0.001), while the differences did not reach statistical significance in recessive and allelic models (p>0.05). (Table 2 and S4 Table, Fig 2)

**Table 2:** Meta-analysis results for the selected IL-1 gene cluster polymorphisms.

	*Dominant genetic model*^a^		*Recessive genetic model*^b^		*Allelic genetic model*^c^	
	OR, 95% CI	Z; P value	OR, 95% CI	Z; P value	OR, 95% CI	Z; P value
***IL1A* polymorphisms**						
**-889 C>T (rs1800587)**						
Pooled	1.15, 0.90–1.49	1.11; 0.27	1.19, 0.65–2.16	0.57; 0.57	1.13, 0.90–1.42	1.08; 0.28
European Ancestry	1.14, 0.56–2.32	0.37; 0.71	-	-	-	-
North Indian Ancestry	1.16, 0.88–1.52	1.04; 0.30	1.19, 0.65–2.16	0.57; 0.57	1.13, 0.90–1.42	1.08; 0.28
*Subgroup differences*	*I*^*2*^ *=* 0%; P_Q_ = 0.98		*I*^*2*^ *=* NA; P_Q_ = NA		*I*^*2*^ *=* NA; P_Q_ = NA	
**+4845 G>T (rs17561)**						
Pooled	0.75, 0.41–1.35^R^	0.96; 0.34^R^	1.03, 0.67–1.57	0.13; 0.90	1.03, 0.85–1.24	0.28; 0.78
European Ancestry	0.99, 0.73–1.33	0.08; 0.94	1.08, 0.69–1.70	0.34; 0.73	1.01, 0.81–1.27	0.12; 0.91
Middle Eastern Ancestry	0.33, 0.19–0.59	3.77; 0.0002*	-	-	-	-
North Indian Ancestry	1.11, 0.76–1.61	0.53; 0.59	0.70, 0.20–2.43	0.56; 0.58	1.06, 0.75–1.49	0.33, 0.74
*Subgroup differences*	*I*^*2*^ *=* 84.8%; P_Q_ = 0.001		*I*^*2*^ *=* 0%; P_Q_ = 0.52		*I*^*2*^ *=* 0%; P_Q_ = 0.83	
***IL1B* polymorphisms**						
**-511 C>T (rs16944)**						
Pooled	0.87, 0.75–1.00^R^	1.90; 0.06^R^	0.85, 0.69–1.04^R^	1.55; 0.12^R^	0.94, 0.84–1.05^R^	1.06; 0.29^R^
European Ancestry	0.97, 0.83–1.12	0.45; 0.65	0.92, 0.64–1.33^R^	0.43; 0.67^R^	1.02, 0.87–1.20^R^	0.25; 0.80^R^
Middle Eastern Ancestry	0.89, 0.70–1.12	1.02; 0.31	0.84, 0.63–1.11	1.24; 0.22	0.89, 0.76–1.05	1.33; 0.18
Asian Ancestry	0.54, 0.28–1.05	1.83; 0.07	-	-	-	-
North Indian Ancestry	0.73, 0.54–0.99	2.05; 0.04*	0.62, 0.39–0.98	2.03; 0.04*	0.76, 0.61–0.94	2.50; 0.01*
African Ancestry	0.45, 0.25–0.83	2.54; 0.01*	0.90, 0.50–1.59	0.38; 0.71	0.73, 0.51–1.03	1.78; 0.07
*Subgroup differences*	*I*^*2*^ *=* 54.5%; P_Q_ = 0.07		*I*^*2*^ *=* 0%; P_Q_ = 0.60		*I*^*2*^ *=* 51.3%; P_Q_ = 0.10	
**-1903 C>T (rs1143627)**						
Pooled	0.95, 0.74–1.23	0.37; 0.71	0.93, 0.66–1.31	0.44; 0.66	0.96, 0.80–1.14	0.49; 0.62
European Ancestry	1.06, 0.67–1.68	0.25; 0.80	0.96, 0.51–1.82	0.11, 0.91	1.02, 0.73–1.43	0.12, 0.90
North Indian Ancestry	0.91, 0.68–1.23	0.60; 0.55	0.91, 0.60–1.37	0.45; 0.66	0.93, 0.75–1.15	0.66, 0.51
*Subgroup differences*	*I*^*2*^ *=* 0%; P_Q_ = 0.59		*I*^*2*^ *=* 0%; P_Q_ = 0.88		*I*^*2*^ *=* 0%; P_Q_ = 0.65	
**-3954 C>T (rs1143634)**						
Pooled	1.05, 0.92–1.20	0.76; 0.44	1.02, 0.75–1.38	0.10; 0.92	1.04, 0.93–1.17	0.73; 0.47
European Ancestry	1.12, 0.93–1.35	1.19; 0.23	1.03, 0.67–1.59	0.15; 0.88	1.08, 0.92–1.27	0.99, 0.32
Middle Eastern Ancestry	1.00, 0.81–1.25	0.02; 0.98	1.09, 0.67–1.78	0.34; 0.73	1.03, 0.85–1.25	0.30, 0.77
North Indian Ancestry	0.98, 0.72–1.33	0.16; 0.87	0.76, 0.31–1.85	0.61; 0.54	0.96, 0.73–1.25	0.33, 0.75
*Subgroup differences*	*I*^*2*^ *=* 0%; P_Q_ = 0.65		*I*^*2*^ *=* 0%; P_Q_ = 0.78		*I*^*2*^ *=* 0%; P_Q_ = 0.72	
**-5887 C>T (rs1143633)**						
Pooled	0.96, 0.78–1.19	0.35; 0.72	0.93, 0.66–1.32	0.39; 0.69	0.96, 0.82–1.13	0.46; 0.65
European Ancestry	0.89, 0.66–1.20	0.77; 0.44	0.85, 0.54–1.34	0.69; 0.49	0.90, 0.72–1.13	0.91; 0.36
North Indian Ancestry	1.04, 0.77–1.40	0.26; 0.80	1.06, 0.62–1.79	0.20; 0.84	1.04, 0.82–1.31	0.30; 0.77
*Subgroup differences*	*I*^*2*^ *=* 0%; P_Q_ = 0.46		*I*^*2*^ *=* 0%; P_Q_ = 0.55		*I*^*2*^ *=* 0%; P_Q_ = 0.40	
***IL1RN* polymorphisms**						
**86bp VNTR (PMID 14563376)**						
Pooled	0.96, 0.81–1.14^R^	0.49; 0.62^R^	0.93, 0.69–1.26^R^	0.46; 0.65^R^	1.01, 0.86–1.18^R^	0.08; 0.94^R^
European Ancestry	0.93, 0.79–1.10	0.83; 0.40	0.81, 0.60–1.09	1.40; 0.16	1.01, 0.83–1.22^R^	0.10; 0.92^R^
Middle Eastern Ancestry	1.05, 0.85–1.31	0.46; 0.64	1.09, 0.74–1.60	0.43; 0.66	1.01, 0.84–1.21	0.09; 0.93
North Indian Ancestry	1.51, 1.03–2.23	2.10; 0.04*	2.25, 1.09–4.64	2.19; 0.03*	1.62, 1.16–2.27	2.85; 0.004*
Mixed Ancestry	0.58, 0.41–0.82	3.13; 0.002*	0.68, 0.41–1.13	1.49; 0.14	0.66, 0.51–0.85	3.18; 0.001*
*Subgroup differences*	*I*^*2*^ *=* 79%; P_Q_ = 0.003		*I*^*2*^ *=* 62.4%; P_Q_ = 0.05		*I*^*2*^ *=* 83.4%; P_Q_ = 0.0004	
**+8006 T>C (rs419598)**						
Pooled	0.97, 0.68–1.39^R^	0.17; 0.87^R^	0.98, 0.72–1.32	0.16; 0.87	0.98, 0.76–1.26^R^	0.15; 0.88^R^
European Ancestry	1.21, 1.01–1.45	2.08; 0.04*	0.99, 0.68–1.43	0.06; 0.96	1.12, 0.95–1.33	1.34; 0.18
North Indian Ancestry	1.06, 0.68–1.66	0.26; 0.79	1.24, 0.25–6.19	0.26; 0.79	1.07, 0.70-.163	0.31; 0.75
Mixed Ancestry	0.58, 0.41–0.81	3.14; 0.002*	0.92, 0.52–1.61	0.31; 0.76	0.70, 0.54–0.92	2.60; 0.009*
*Subgroup differences*	*I*^*2*^ *=* 85.8%; P_Q_ = 0.0009		*I*^*2*^ *=* 0%; P_Q_ = 0.93		*I*^*2*^ *=* 76.8%; P_Q_ = 0.01	

OR, 95% CI: Odds Ratio with its 95% Confidence Interval; ^R^: Results derived using Random effects for analysis. Fixed effects were used for all other calculations; P_Q_: Cochran’s Q statistics; *I*^*2*^: Higgin’s *I*^*2*^ statistics.

* A p value of <0.05 was considered as statistically significant.

^a^ Dominant genetic model: *TT+CT* vs. *CC* for A-889 C>T; *TT+GT* vs. *GG* for A+4845 G>T; *TT+CT* vs. *CC* for B-511 C>T; *TT+CT* vs. *CC* for B-1903 C>T; *TT+CT* vs. *CC* for B-3954 C>T; *TT+CT* vs. *CC* for B-5887 C>T; *2/2+X/2* vs. *X/X* for RN 86bp VNTR (*X* = Any other allele than *allele 2*); *CC+CT* vs. *TT* for RN+8006 T>C.

^b^ Recessive genetic model: *TT* vs. *CT+CC* for A-889 C>T; *TT* vs. *CT+GG* for A+4845 G>T; *TT* vs. *CT+CC* for B-511 C>T; *TT* vs. *CT+CC* for B-1903 C>T; *TT* vs. *CT+CC* for B-3954 C>T; *TT* vs. *CT+CC* for B-5887 C>T; *2/2* vs. *X/2+X/X* for RN 86bp VNTR; *CC* vs. *CT+TT* for RN+8006 T>C.

^c^ Allelic genetic model: *Allele T* vs. *Allele C* for A-889 C>T; *Allele T* vs. *Allele G* for A+4845 G>T; *Allele T* vs. *Allele C* for B-511 C>T; *Allele T* vs. *Allele C* for B-1903 C>T; *Allele T* vs. *Allele C* for B-3954 C>T; *Allele T* vs. *Allele C* for B-5887 C>T; *Allele 2* vs. *Allele X* for RN 86bp VNTR; *Allele C* vs. *Allele T* for RN+8006 T>C.

**Fig 3:**
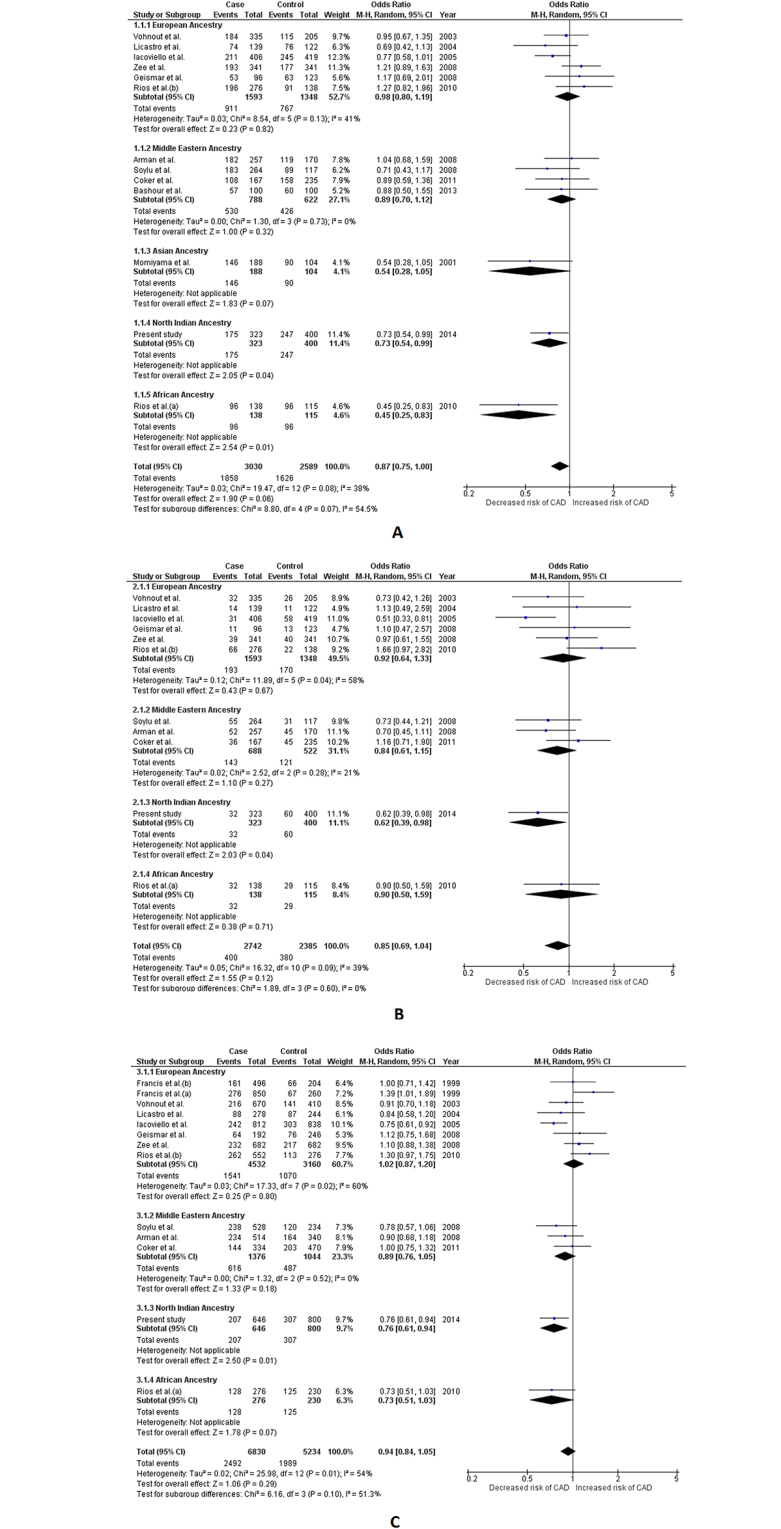
Forest plots depicting meta-analysis results for *IL1B* -511 C>T (rs16944) polymorphism. Panel A: Effect size estimation using dominant genetic model (*TT+CT vs*. *CC*); Panel B: Effect size estimation using recessive genetic model (*TT vs*. *CT+CC*); Panel C: Effect size estimation using allelic genetic model (*Allele T vs*. *Allele C*). Pooled effect size estimates for dominant, recessive and allelic genetic models were obtained using random effects for analysis. Random effects were also used to calculate effect size estimates for European Ancestry group in recessive and allelic genetic model. Fixed effects were used for dominant genetic model in the European Ancestry group, as well as all ancestral groups in all three genetic models. Revised effect size estimates using fixed effects are given in Table 2.

A total of 4 SNPs from the *IL1B* gene, viz. -511 C>T, -5887 C>T, -3954 C>T and -1903 C>T, were selected for the present meta-analysis. Fifteen different studies (including ours’) were included for -511 C>T, with a total sample of 7,429 (4,376 cases and 3,053 controls) and the quantitative synthesis was carried out using appropriate effects for analysis.[26–37] Significant heterogeneity in the group compelled us to use random effects for the pooled analysis. Although none of the three genetic models reached the required significance level to confirm association (p> 0.05), the results from the dominant model did depict a trend, which suggested a protective nature of this SNP against CAD (OR = 0.87, 95% CI = 0.75–1.00, Z value = 1.90 and p = 0.06). Association amongst ancestral subgroups was also tested employing appropriate effects for analysis. Protective effect of the -511 C>T polymorphism was seen more or less amongst all genetic models of AA, NIA and AFA subgroups. All three genetic models amongst NIA (p≤ 0.04) and the dominant model of AFA (p< 0.01) reached the required statistical significance to confirm association with CAD., On the other hand dominant model amongst AA and allelic model amongst AFA could not reach the desired statistical significance but managed to show a clear trend of association with CAD (p = 0.07 for each). Contrastingly, no hint of association was seen amongst EA and MEA subgroups (p> 0.05). This contrast of association statuses amongst different ancestral subgroups resulted in significant subgroup differences which was clearly seen in dominant and allelic genetic models (*I*^*2*^ = 54.5%, P_Q_ = 0.07 and *I*^*2*^ = 51.3%; P_Q_ = 0.10 respectively). (Table 2 and S4 Table, Fig 3)

Including the present study, two studies[38] were included for -1903 C>T (total sample: 1,041 with 524 cases and 517 controls), 9 studies[25–27, 31, 32, 34–36] for -3954 C>T (total sample: 3,975 with 2,018 cases and 1,957 controls) and 2 studies[26] for -5887 C>T (total sample: 1,405 with 664 cases and 741 controls). Their data was meta-analyzed employing all three genetic models using appropriate effects for analysis. No hint of association was deduced in the pooled analysis vide any of the three genetic models for any of the three aforementioned SNPs (p value range = 0.62–0.71 for -1903 C>T, p value range = 0.44–0.92 for -3954 C>T and p value range = 0.65–0.72 for -5887 C>T). Additionally, none of the ancestral subgroups for these three SNPs showed any hint association with CAD either (p> 0.05). Also, no significant subgroup differences was detected in case of these aforementioned 3 *IL1B* SNPs (*I*^*2*^< 50% and P_Q_> 0.10). (Table 2 and S4 Table, S2–S4 Figs)

**Fig 4:**
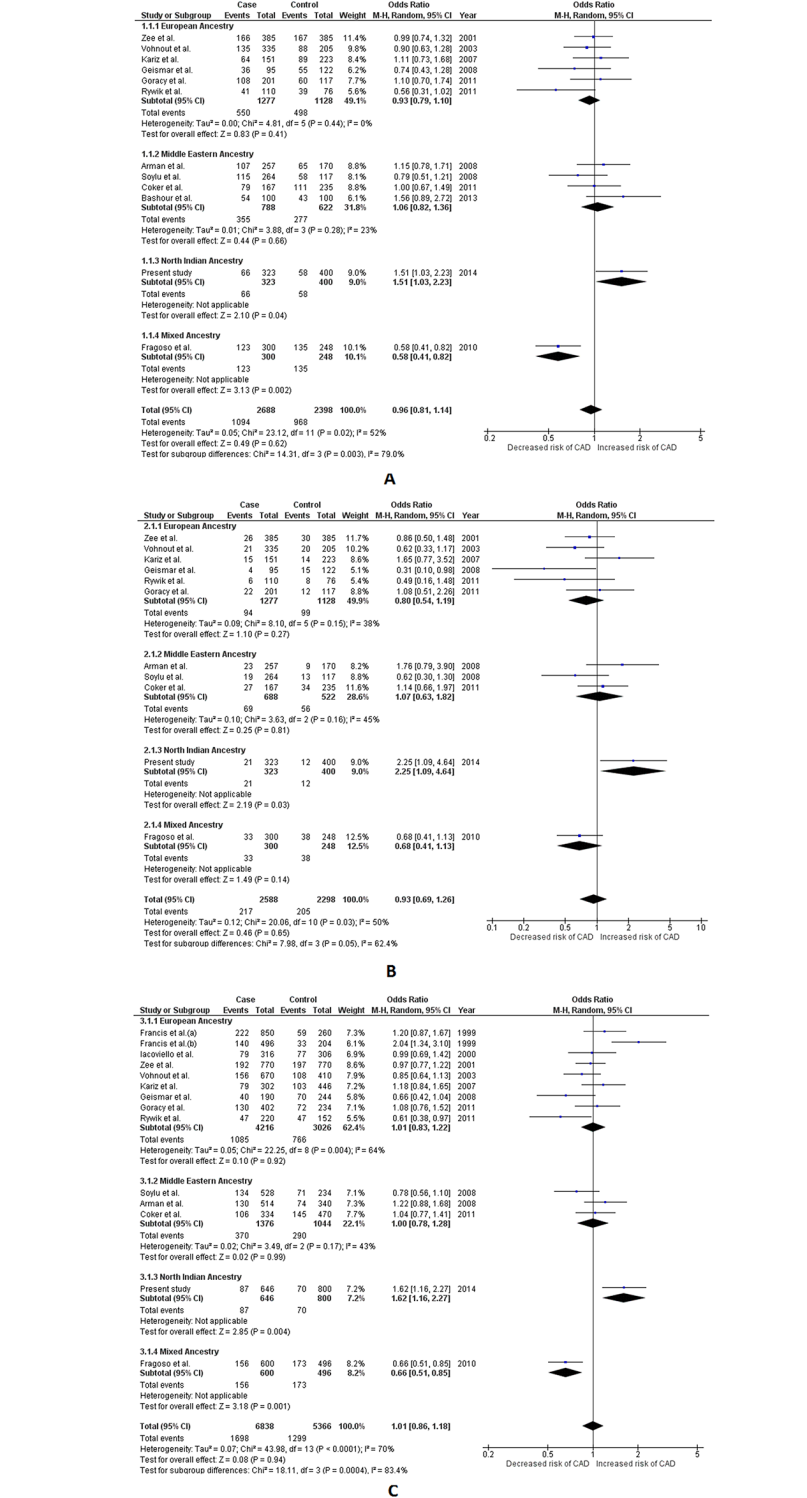
Forest plots depicting meta-analysis results for *IL1RN* 86bp VNTR (PMID 14563376) polymorphism. Panel A: Effect size estimation using dominant genetic model (*2/2+X/2 vs*. *X/X*); Panel B: Effect size estimation using recessive genetic model (*2/2 vs*. *X/2+X/X*); Panel C: Effect size estimation using allelic genetic model (*Allele 2 vs*. *Allele X*). Pooled effect size estimates for dominant, recessive and allelic genetic models were obtained using random effects for analysis. Random effects were also used to calculate effect size estimates for European Ancestry group in allelic genetic model. Fixed effects were used for dominant and recessive genetic model in the European Ancestry group, as well as all ancestral groups in all three genetic models. Revised effect size estimates using fixed effects are given in Table 2. Comparisons were performed according to “allele 2” and “allele X” nomenclature where allele X is defined here as any other allele, than allele 2.

Two common polymorphisms, viz. 86bp VNTR and +8006 T>C, were selected for meta-analysis from the *IL1RN* gene. Including the present study, a total of 15 different studies[27–29, 32, 34–36, 38–43] were included for 86bp VNTR with a total sample of 6,302 (3,519 cases and 2,783 controls). Appropriate effects were employed for quantitative synthesis. The comparisons for 86bp VNTR were performed using our previously defined X/X, X/2 and 2/2 nomenclature. The pooled results obtained using random effects for analysis did not show any hint with association for 86bp VNTR polymorphism with CAD (p value range = 0.62–0.94 for all three genetic models). Subgroup analysis also revealed lack of association among EA and MEA subgroups (p>0.05). However, both the dominant and recessive genetic models of the NIA subgroup showed statistically significant association, where the genotypes carrying ≥1 minor allele (allele 2) were found to be significantly associated with an increased risk for CAD (OR = 1.51, 95%CI = 1.03–2.23, Z value = 2.10, p = 0.04 and OR = 2.25, 95%CI = 1.09–4.64, Z value = 2.19, p = 0.03 for dominant and recessive model respectively). The allele 2 in the NIA subgroup also demonstrated to be independently associated with higher odds for CAD (OR = 1.62, 95% CI = 1.16–2.27, Z value = 2.85, p = 0.004 for the allelic model). Contrastingly, the results from dominant and allelic models of the MA subgroup suggested that the presence of allele 2 in a genotype can confer significant protection against CAD (OR = 0.58, 95% CI = 0.41–0.82, Z value = 3.13, p = 0.002 and OR = 0.66, 95% CI = 0.51–0.85, Z value = 3.18, p = 0.001 for dominant and allelic model respectively). The results for the recessive model of the MA subgroup, however did not reach the required statistical significance (p> 0.05). Contrasting results in the ancestral subgroups resulted in significant subgroup differences which was detectible in all three assessed genetic models (*I*^*2*^ = 79%, P_Q_ = 0.003 in dominant *I*^*2*^ = 62.4%, P_Q_ = 0.05 in recessive and *I*^*2*^ = 83.4%; P_Q_ = 0.0004 in allelic genetic model). (Table 2 and S4 Table, Fig 4)

**Fig 5:**
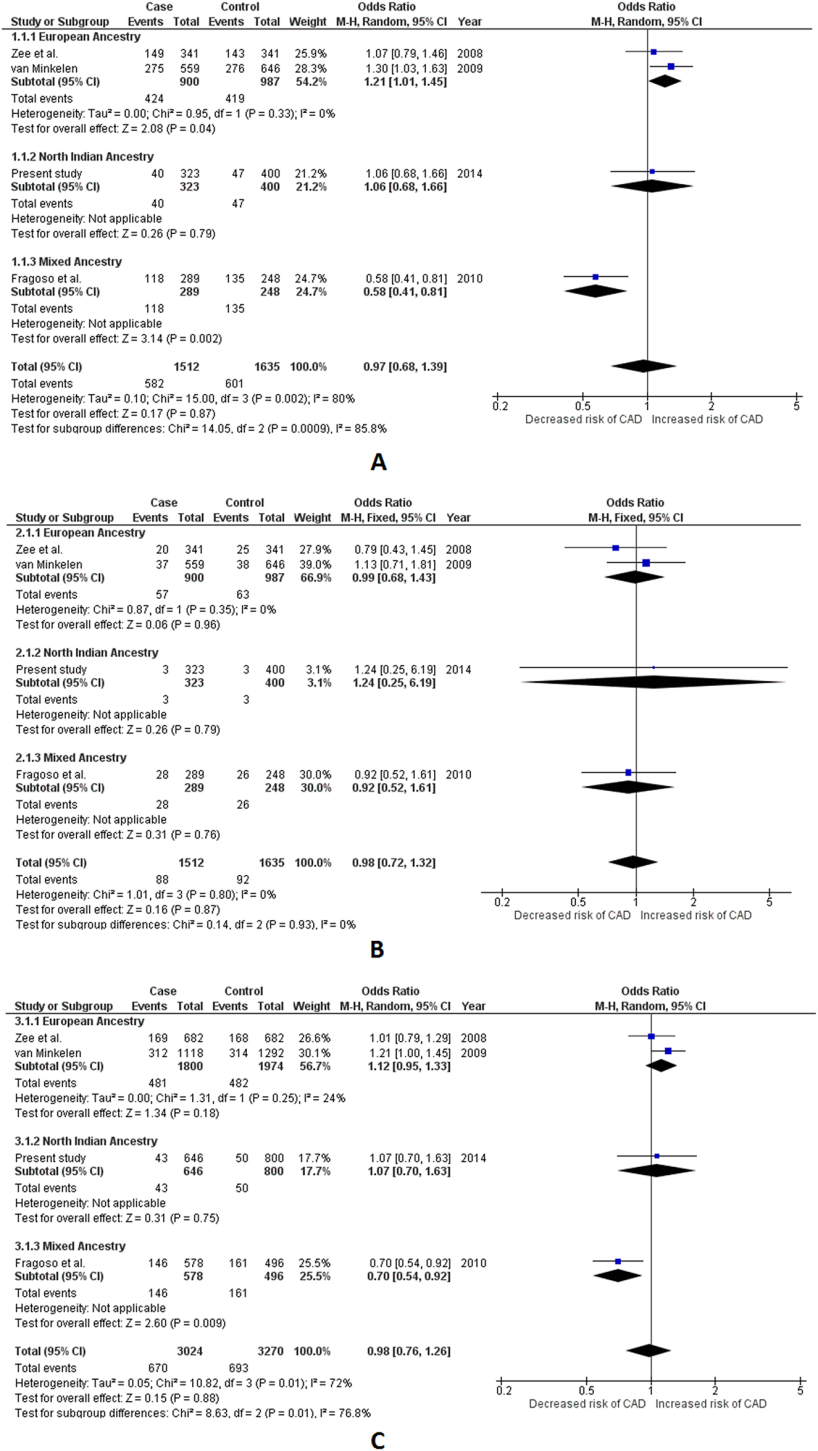
Forest plots depicting meta-analysis results for *IL1RN* +8006 T>C (rs419598) polymorphism. Panel A: Effect size estimation using dominant genetic model (*CC+CT vs*. *TT*); Panel B: Effect size estimation using recessive genetic model (*CC vs*. *CT+TT*); Panel C: Effect size estimation using allelic genetic model (*Allele C vs*. *Allele T*). Pooled effect size estimates for dominant, recessive and allelic genetic models were obtained using random effects for analysis. Fixed effects were used for all ancestral groups in all three genetic models. Revised effect size estimates using fixed effects are given in Table 2.

Four studies[26, 42, 44] were included for meta-analysis for +8006 T>C with a total sample of 3,147 (1,512 cases/1,635 controls). Based on the degree of heterogeneity detected, appropriate effects were used for both pooled as well as subgroup analysis. No hint of association of +8006 T>C polymorphism with CAD was deduced in the pooled analysis (p> 0.05 for all three genetic models). The subgroup results were however interesting. The results in the dominant model of the EA subgroup suggested that the presence of ≥1 “C” (minor) allele in a genotype significantly increases the risk of CAD (OR = 1.21, 95% CI = 1.01–1.45, Z value = 2.08, p value = 0.04), however the recessive and allelic models of EA indicated lack of such association (p> 0.05). While the NIA subgroup remained non-associated with CAD (p>0.05 in all three genetic models), the MA subgroup suggested significant protective nature of the minor allele at least in the dominant and the allelic model (OR = 0.58, 95% CI = 0.41–0.81, Z value = 3.14, p = 0.002 and OR = 0.70, 95% CI = 0.54–0.92, Z value = 2.60, p = 0.009 respectively). This contrast among ancestral subgroups resulted in significant subgroup differences in the dominant as well as the allelic model (*I*^*2*^ = 85.8%, P_Q_ = 0.0009 and *I*^*2*^ = 76.8%, P_Q_ = 0.01 respectively). (Table 2 and S4 Table, Fig 5)

### Publication bias assessment

Publication bias was assessed for each SNP, in its each genetic model (separately for pooled and its major ancestral subgroups) which had ≥3 included studies using two statistical methods viz. Begg’s funnel plot test[23] and Egger’s test.[24] Using our preset criteria, only 4 SNPs viz. B-511 C>T, B-3954 C>T, RN86bp VNTR and RN+8006 T>C, qualified for publication bias assessment. Funnel plots for each qualifying SNP and its each genetic model displaying Egger’s estimates (for pooled and major ancestral groups) are given as supplementary figures. S5 Fig displays all three funnel plots for *IL1B* -511 C>T and *IL1B* -3954 C>T, polymorphisms, whereas S6 Fig displays all three funnel plots for *IL1RN* 86bp VNTR and *IL1RN* +8006 T>C polymorphisms. Each point in these plots represents the OR of a study plotted against the standard error (SE) of this aforementioned OR. Studies belonging to different ancestral subgroups have different indicators in these plots and they seem to be generally contained within the inverted cone, indicating no significant publication bias, at least in the pooled analysis. However, Egger’s test which although detected no significant publication bias for most the groups/subgroups, did manage to produce some significant p values in a few groups/subgroups. Subgrouping on the basis of ancestry did successfully tone down the observed significant p values seen in the pooled analysis, indicating heterogeneity as the culprit. Although, all the groups with significant Egger’s p value were small and the culprit could have been inherent heterogeneity, no significant Egger’s p values were detected in the groups/subgroups which were significantly associated with the disease. (Table 2, S5 and S6 Figs)

### Sensitivity analysis

Sensitivity analysis was performed in each study group, where we excluded studies one after another and conducted the analysis after each omission (in groups of ≥ 5 studies). Only three SNPs viz. B-511 C>T, B-3954 C>T, RN86bp VNTR qualified for the same. The summary effects deduced for these SNP’s (among all of the studied genetic model groups) did not alter significantly i.e. from non-association to significant association or the other way around, indicating the robustness of the present meta-analysis.

## Discussion

The present association study is the largest so far ever conducted amongst ethnic North Indians both in terms of the sample size assessed and the number of IL-1 gene cluster variants studied. Power analysis was also conducted using the generated data for all 11 SNPs. Our power analysis indicated that the sample size of our case-control study was sufficient to support the derived results (Power = 0.9900–0.9999 for all 11 SNPs). Our updated meta-analysis is the most comprehensive on the subject presented so far. It has yielded novel yet reproducible insight into the association statuses of IL-1 gene cluster variants with CAD amongst diverse ancestral subgroups. The first and foremost merit of the present meta-analysis is that it has been conducted ensuring strict adherence to all contemporary recommendations and guidelines. It can be considered far superior to the only published meta-analysis on this subject,[10] both in terms of volume of studies included and number of SNPs assessed. Ancestral stratification of studies was also not done rigorously in the meta-analysis by Zhou *et al*.[10]; a mistake which has been duly rectified in our present meta-analysis. It is well known that association of genetic variants with multifactorial diseases (such as CAD), can be influenced by the interplay of various environmental factors. Their effects on exposed populations can ultimately lead to ancestral differences in association statuses. This phenomenon was also duly attested by the results of our subgroup analysis where at instances the same gene variant showed non-association in one ancestral subgroup while showing significant association in the other ancestral subgroup. Difference in terms of association statuses was also seen for several SNPs, where the same variant proved to be risk factor for CAD in one ancestral subgroup while acting as a protective factor for the other. Our subgroup analysis thus helped us to produce novel insights, imparting substantial merit to the present meta-analysis. We thus believe our study has successfully achieved both the aims laid out at the outset and is statistically powered to stand by its obtained results.

### *IL1A* gene variants and CAD

Our case-control study suggested lack of association of both the studied *IL1A* gene SNPs (i.e. -889 C>T and +4845 G>T) with CAD. Neither of the possible haplotypes constructed out of the two SNPs showed any significant association. Neither pooled nor our subgroup analysis deduced any significant associations for -889 C>T. Such result was expected, as none of the evidence published so far indicated any hint of association. Non association with restenosis after percutaneous transluminal coronary angioplasty (PTCA) has also been reported for -889 C>T which probably testifies its lack of association with the disease.[45] No published data reporting the association of -889 C>T polymorphism with levels of systemic/cardio-specific inflammatory markers exist in public domain.

Only one study so far, (amongst Middle Easterners) has reported significant association of +4845 G>T with CAD in a relatively small sample size of 200 (100 cases and 100 controls).[27] Its contribution however proved to be insufficient to effect any change in our pooled results. The aforementioned study,[27] however effected significant association in the dominant model of MEA. Clear evidence indicating non-association of +4845 G>T polymorphism with levels of systemic inflammatory activation markers (C-reactive protein: CRP and IL-1Ra), soluble endothelial activation markers (vonWillebrand factor: vWF and E-selectin) and Troponin T levels has been reported from a cohort of 63 American patients presenting with non-ST-elevation ACS (p >0.05).[46] A similar study amongst a cohort of Danish patients, also failed to associate this SNP with CAD (and periodontitis) or any of its systemic inflammatory markers.[32] Since the association reported by Bashour *et al*.[27] lacks biological plausibility, we cannot comment on the possible underlying mechanism.

Absence of any relevant and statistically significant published data indicates non-functional nature of both these *IL1A* SNPs. This explains their lack of association seen in our case-control study and the present meta-analysis.

### *IL1B* gene variants and CAD

Although none of the association tests for all 4 *IL1B* SNPs (i.e. -511 C>T, -1903 C>T, -3954 C>T and -5887 C>T), in the present case-control study yielded desired statistical significance to confirm association, distinct trends suggesting possible association were seen for -511 C>T. Congruent with the results of our case-control study, subgroup analysis for -511 C>T hinted associations for certain ancestral subgroups (AA), and even attested the same for the NIA subgroup, vide all three genetic models. Results of our case-control study as well as our meta-analysis suggested that C to T substitution at position 511bp of the *IL1B* gene might act as protective factor against CAD, at least amongst subjects of North Indian Ancestry. Over the years most association studies amongst subjects belonging varied ancestries,[26–30, 32–37] as well as the only published meta-analysis on this subject[10] have failed to report any significant association of -511 C>T with CAD. However, similar to the association trend seen amongst our study cohort of NIA, a couple of other studies (amongst Europeans), have also implicated “T” allele and “CT” genotype as a protective factor against CAD. [31, 33] This trend of association amongst NIA was also verified by the statistically significant NIA subgroup results obtained in our meta-analysis. The biochemical basis of such association is rather intriguing. Since non association of any of the *IL1B* -511 genotypes with CRP values has already been established,[47] the protective nature of -511 C>T polymorphism seen against CAD, probably suggests the involvement of a different biochemical mechanism. It is already proven that IL-1β is a major link between inflammation and coagulation as it is able to stimulate the synthesis of tissue factor (TF) from monocytes and endothelial cells.[48, 49] Further explanation of the link between the -511C>T genotypes and coronary thrombosis has been suggested by Iacoviello and coworkers.[31] They found that blood mononuclear cells from “protective” T allele carriers, are also “protected” from pro-active coagulation in response to an inflammatory stimulus.[31] The release of IL-1β on lipopolysaccharide (LPS) stimulation among “T” allele carriers was also found to be significantly lower than that from mononuclear cells in “C” allele carriers. [31] Mononuclear cells in these T allele carriers also expressed a significantly lower amount of TF procoagulant activity after LPS stimulation.[31] This specific inhibition of TF expression by monoclonal antibodies against IL-1β,[31] confirms the relevance of endogenous IL-1β in stimulating the expression of TF on cell surface. This explains how “C” allele carriers have an enhanced risk of thrombotic events amongst which the cellular response to inflammatory stimuli is modulated which subsequently promotes blood clotting. Alternatively, presence of a “T” allele in an individual may impart a decreased susceptibility to cardiac events through a decreased inflammatory response leading to a decreased activation of blood coagulation.

Lack of associations for -1903 C>T, -3954 C>T and -5887 C>T with CAD, as seen in our case-control study were attested by both the pooled as well as subgroup results of our meta-analysis. Over the last decade, these aforementioned *IL1B* SNPs have been extensively investigated with respect to CAD/MI, yielding negative results.[25–27, 31, 32, 34–36, 38] Lack of association for -3954 C>T polymorphism with CAD has even been attested before, vide a published meta-analysis[10]. Since we failed to discover any substantial evidence advocating association, it is safe to say that these SNPs may have little or no role in the pathogenesis of atherosclerotic heart disease.

Our case-control study also identified several *IL1B* haplotypes that might impart diverse effects on the outcome. The underlying biochemical mechanisms behind these diverse effects are unknown and warrant functional studies.

### *IL1RN* gene variants and CAD

No hint of association was seen when we compared genotypic and allelic frequencies in cases with that in controls for 4 out of 5 selected *IL1RN* gene polymorphisms (i.e. +9589 A>T, +8006 T>C, +8061 C>T and +111000 T>C) in our present case-control study. Association status tested for *IL1RN* 86bp VNTR with CAD, both in our case-control study as well as in the pooled results our meta-analysis also did not reach the desired statistical significance. These results are in concordance with results of only published meta-analysis on this subject.[10] However, comparisons according to allele 2 and allele X nomenclature, yielded promising trends of association in our case-control study which was further validated by our meta-analysis where significant association was seen amongst the NIA subgroup. It seems that the presence allele 2 tends to increase the risk of CAD, at least in subjects of North Indian ancestry. Contrasting trend of association was observed in MA subgroup, where presence of allele 2 seemed to impart protection against CAD (at least in the dominant and allelic genetic model). Such result suggests that this SNP behaves in different ways in populations from different ancestral backgrounds. Such contrasting effect of this SNP has also been previously reported. While the allele 2 showed its protective nature in a couple of studies,[42, 43] it also has been implicated as a risk factor for CAD in one published study.[28] Latkovskis *et al*.,[47] reported that presence of allele 2 in a genotype is associated with lower CRP values which can explain its protective nature seen amongst several ethnic populations.[42, 43] Genetic variation in different ethnic populations coupled with different environmental factors may account for contrasting results seen the resultant phenotypes. Unidentified gene-gene or gene-environment interactions could thus very well be held responsible. We can only speculate about the pathway to explain such contrasting associations, but since allele 2 has been shown to be associated with an enhanced IL-1β production, (at least in-vitro)[50] it is possible that it can add on to the overall risk of CAD amongst the carriers of this allele as seen in the our study cohort belonging to NIA. We believe that more functional studies are warranted in order explain the clear biological basis of such dissimilar associations seen among various ethnic populations.

Our meta-analysis although validated the lack of association for +8006 T>C in the pooled analysis, but discovered significant yet contrasting association trends in a couple of ancestral subgroups (p<0.05). While the dominant model of the EA subgroup suggested this SNP as a risk factor for CAD, the dominant and allelic model for the MA subgroup suggested it to be protective factor. Published literature does not explain the underlying biological basis of such contrasting associations and thus probably requires further investigation.

Our pooled result also concurs with the CARDIoGRAMplusC4D GWAS data, which incidentally is the most comprehensive meta-analysis of published GWAS studies on the subject.[51] Severe ancestral diversity was however seen in terms of association with CAD, for at least four SNPs residing in the IL-1 gene cluster (i.e. for *IL1A* +4845 G>T, *IL1B* -511 C>T, *IL1RN* +8006 T>C and *IL1RN* 86bp VNTR) which duly qualifies to be a highlight of the present meta-analysis.

Every genetic association study and their meta-analyses for a particular disease/condition have their potential limitations. The clinical utility of association studies like ours’ is always in question and the derived significant p values should be always be accompanied by biological plausibility of the seen association. In the present case-control study the two SNPs hinting association with the disease (i.e. *IL1B*-511C>T and *ILIRN* 86bp VNTR) have previously been thoroughly (if not conclusively) investigated with respect to CAD. Also, their biological plausibility has at least been previously indicated, if not established. The fact that this aforementioned plausibility is not yet conclusively established may qualify to be a primary limitation of our study. Also since very few published reports are available from our region, and since it’s a land of significant genetic diversity, we are not sure if our results would hold true in other population pockets of India. More association studies among cohorts belonging to North Indian ancestry are thus warranted in order to establish/negate these seen associations. Ideally, association among serum levels of interleukins with their genotypes should also have been investigated in the present case-control study, however arranging the logistics of same in a multicentric study, like ours’ was difficult, and this qualifies to be another major limitation. Possibility of errors in genotyping, presence of selection bias and risk of inadequate sample size amongst different studies could easily qualify to be listed as limitations in every meta-analysis like ours’. Any meta-analyses of association studies cannot inspect interference of linkage disequilibrium and thus cannot measure its overall effect on the seen associations. Although, no significant publication bias was detected amongst our study groups, the role of existing publication bias however cannot be completely ruled out which also qualifies to be listed as another small limitation in our present meta-analysis.

## Conclusions

Although our association study amongst NIA failed to conclusively attest to statistically significant associations for all 11 studied IL-1 gene cluster SNPs with CAD, we found hints suggesting possible association for at least a couple of them (viz. B -511 T>C and RN 86bp VNTR). While the presence of >1, T (minor) allele of *IL1B* -511 in a genotype seemed to provide protection against CAD, the presence of >1, T (major) allele seemed to increase the risk of CAD in our study cohort. Conversely allele 2 (minor allele) and genotype X/2 of *IL1RN* 86bp VNTR polymorphism seemed to increase an individual’s risk for CAD. Several haplotype combinations constructed out of studied SNPs belonging to *IL1B* and *IL1RN* genes also showed varied statistically significant associations with CAD.

Congruent with the results of our association study, the pooled results of our meta-analysis also did not show any statistically significant associations between studied IL-1 gene cluster polymorphisms and CAD. However, significant differences in association statuses amongst studied ancestral groups were seen for *IL1A* +4845 G>T, *IL1B* -511 C>T, *IL1RN* 86bp VNTR and *IL1RN* +8006 T>C. The hints of associations deduced for subjects belonging to NIA in our case-control study for both *IL1B* -511 C>T and *IL1RN* 86bp VNTR were duly validated vide significant p values seen in our subgroup analysis. On the other hand, MA subgroup carrying *IL1B* -511 C>T, *IL1RN* 86bp VNTR and *IL1RN* +8006 T>C polymorphisms seemed to enjoy significant protection against CAD. A few other ancestral groups also showed significant associations vide any one of the three constructed genetic models for a few of the studied SNPs, however these associations did not seem conclusive and do require further validation. Although the practical utility of such results are always questionable, the generated information however has the potential to be used after clinical interpretation.

## Supporting Information

S1 Checklist PRISMA Checklist. (DOC)

S2 Checklist Meta-analysis of Genetic Association Studies Checklist. (DOC)

S1 Data Zipped folder containing “Raw Data” for both the case-control study and the present meta-analysis. (ZIP)

S1 Fig Forest plots depicting meta-analysis results for *IL1A* -889 C>T (rs1800587) polymorphism. Panel A: Effect size estimation using dominant genetic model (*TT+CT vs*. *CC*); Panel B: Effect size estimation using recessive genetic model (*TT vs*. *CT+CC*); Panel C: Effect size estimation using allelic genetic model (*Allele T vs*. *Allele C*). Effect size estimates for dominant, recessive and allelic genetic models (for pooled as well as all ancestral subgroups) were obtained using fixed effects for analysis.
(TIF)

S2 Fig Forest plots depicting meta-analysis results for *IL1B* -5887C>T (rs1143633) polymorphism. Panel A: Effect size estimation using dominant genetic model (*TT+CT vs*. *CC*); Panel B: Effect size estimation using recessive genetic model (*TT vs*. *CT+CC*); Panel C: Effect size estimation using allelic genetic model (*Allele T vs*. *Allele C*). Effect size estimates for dominant, recessive and allelic genetic models (for pooled as well as all ancestral subgroups) were obtained using fixed effects for analysis.
(TIF)

S3 Fig Forest plots depicting meta-analysis results for *IL1B* -3954 C>T (rs1143634) polymorphism. Panel A: Effect size estimation using dominant genetic model (*TT+CT vs*. *CC*); Panel B: Effect size estimation using recessive genetic model (*TT vs*. *CT+CC*); Panel C: Effect size estimation using allelic genetic model (*Allele T vs*. *Allele C*). Effect size estimates for dominant, recessive and allelic genetic models (for pooled as well as all ancestral subgroups) were obtained using fixed effects for analysis.
(TIF)

S4 Fig Forest plots depicting meta-analysis results for *IL1B* -1903C>T (rs1143627) polymorphism. Panel A: Effect size estimation using dominant genetic model (*TT+CT vs*. *CC*); Panel B: Effect size estimation using recessive genetic model (*TT vs*. *CT+CC*); Panel C: Effect size estimation using allelic genetic model (*Allele T vs*. *Allele C*). Effect size estimates for dominant, recessive and allelic genetic models (for pooled as well as all ancestral subgroups) were obtained using fixed effects for analysis.
(TIF)

S5 Fig Publication bias assessment among group of studies assessing *IL1B* polymorphisms. Each point in each figure represents OR of a study plotted against the standard error (SE) its OR. Different indicators of the studies belonging to each ancestral group are used in these plots. Panel A: Begg’s funnel plot with Egger’s estimates for dominant genetic model of *IL1B* -511 C>T polymorphism. Panel B: Begg’s funnel plot with Egger’s estimates for recessive genetic model of *IL1B* -511 C>T polymorphism. Panel C: Begg’s funnel plot with Egger’s estimates for allelic genetic model of *IL1B* -511 C>T polymorphism. Panel D: Begg’s funnel plot with Egger’s estimates for dominant genetic model of *IL1B* -3954 C>T polymorphism. Panel E: Begg’s funnel plot with Egger’s estimates for recessive genetic model of *IL1B* -3954 C>T polymorphism. Panel F: Begg’s funnel plot with Egger’s estimates for allelic genetic model of *IL1B* -3954 C>T polymorphism. Abbreviations- EA: European Ancestry; MEA: Middle Eastern Ancestry.
(TIF)

S6 Fig Publication bias assessment among group of studies assessing *IL1RN* polymorphisms. Each point in each figure represents OR of a study plotted against the standard error (SE) its OR. Different indicators of the studies belonging to each ancestral group are used in these plots. Panel A: Begg’s funnel plot with Egger’s estimates for dominant genetic model of *IL1RN* 86bp VNTR polymorphism. Panel B: Begg’s funnel plot with Egger’s estimates for recessive genetic model of *IL1RN* 86bp VNTR polymorphism. Panel C: Begg’s funnel plot with Egger’s estimates for allelic genetic model of *IL1RN* 86bp VNTR polymorphism. Panel D: Begg’s funnel plot with Egger’s estimates for dominant genetic model of *IL1RN* +8006 T>C polymorphism. Panel E: Begg’s funnel plot with Egger’s estimates for recessive genetic model of *IL1RN* +8006 T>C polymorphism. Panel F: Begg’s funnel plot with Egger’s estimates for allelic genetic model of *IL1RN* +8006 T>C polymorphism. Abbreviations- EA: European Ancestry; MEA: Middle Eastern Ancestry.
(TIF)

S1 File List of articles excluded from the present meta-analysis. (DOC)

S1 Table Baseline characteristics of the study cohort. (DOC)

S2 Table Genotypic and allelic comparisons in the present case-control study. (DOC)

S3 Table Haplotypic distribution among cases and controls. (DOC)

S4 Table Meta-analysis: Basic characteristics of the studied groups. (DOC)
