# Supplementary figures and images for: Interleukin-1 Gene Cluster Polymorphisms and Their Association with Coronary Artery Disease: Separate Evidences from the Largest Case-Control Study amongst North Indians and an Updated Meta-Analysis
Source: PLoS One. 2016 Apr 14;11(4):e0153480. doi: 10.1371/journal.pone.0153480 (PMC4831754; doi:10.1371/journal.pone.0153480)

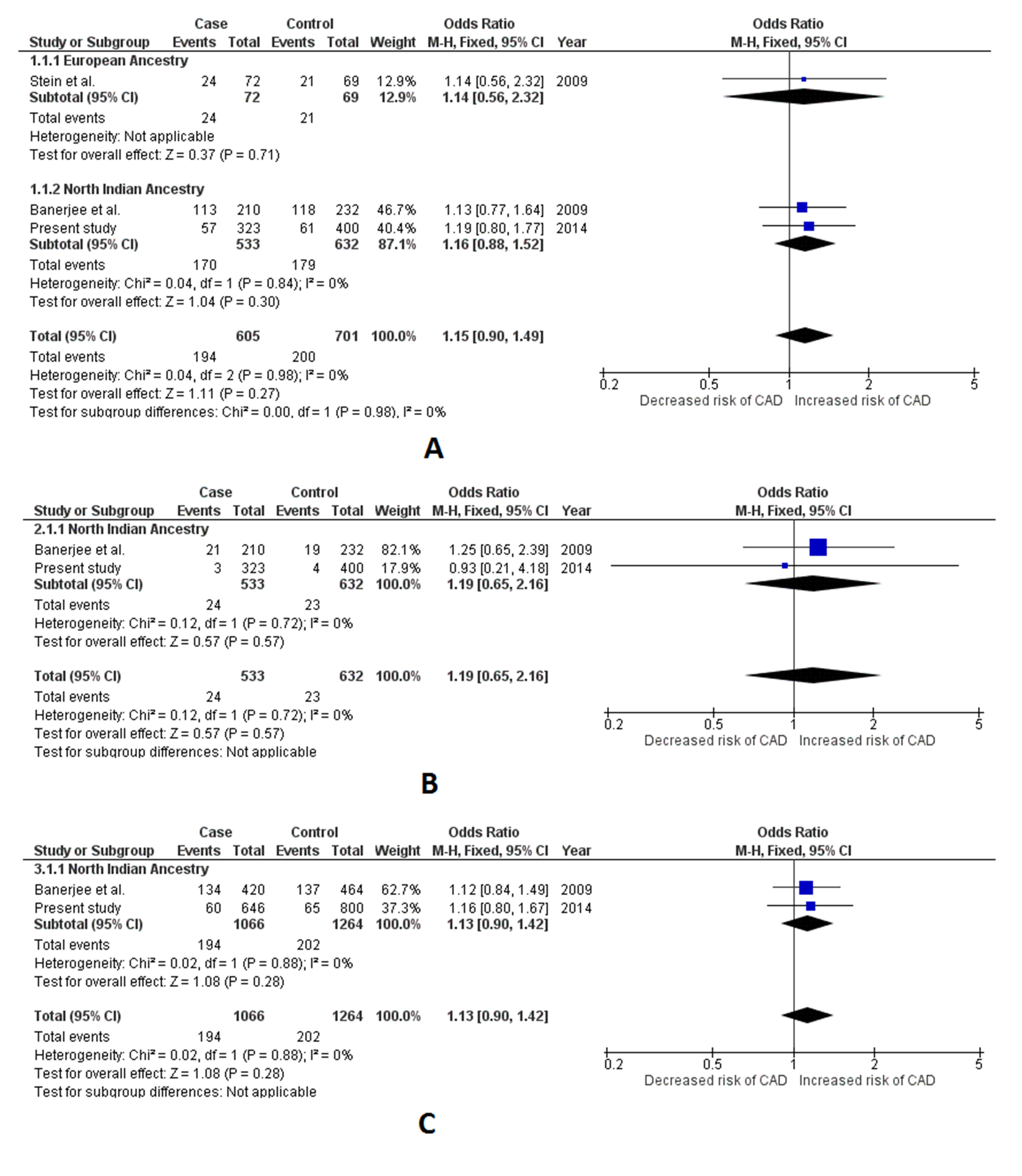

Supplement: S1 Fig — Panel A: Effect size estimation using dominant genetic model (TT+CT vs. CC); Panel B: Effect size estimation using recessive genetic model (TT vs. CT+CC); Panel C: Effect size estimation using allelic genetic model (Allele T vs. Allele C). Effect size estimates for dominant, recessive and allelic genetic models (for pooled as well as all ancestral subgroups) were obtained using fixed effects for analysis. (TIF) [file pone.0153480.s004.tif]

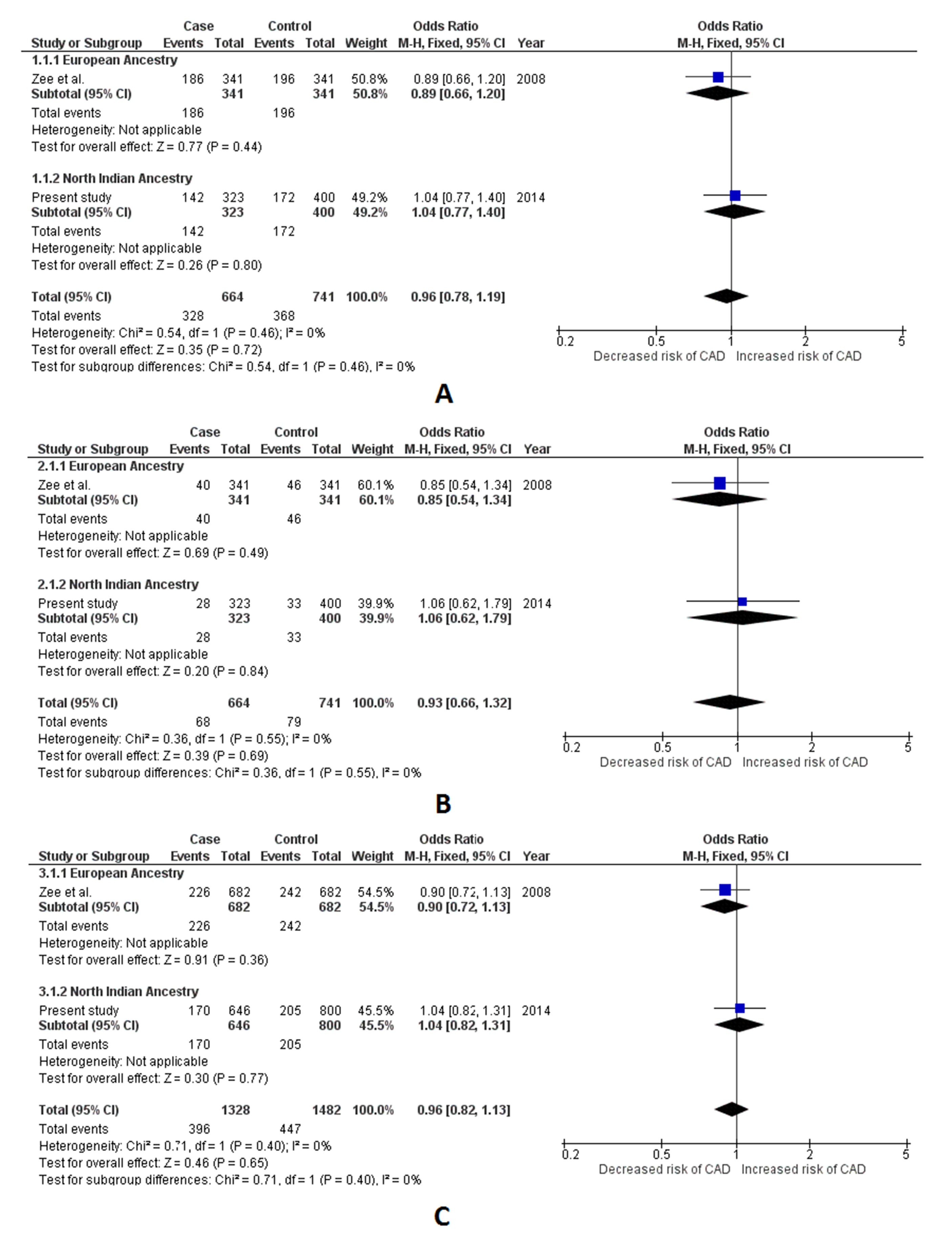

Supplement: S2 Fig — Panel A: Effect size estimation using dominant genetic model (TT+CT vs. CC); Panel B: Effect size estimation using recessive genetic model (TT vs. CT+CC); Panel C: Effect size estimation using allelic genetic model (Allele T vs. Allele C). Effect size estimates for dominant, recessive and allelic genetic models (for pooled as well as all ancestral subgroups) were obtained using fixed effects for analysis. (TIF) [file pone.0153480.s005.tif]

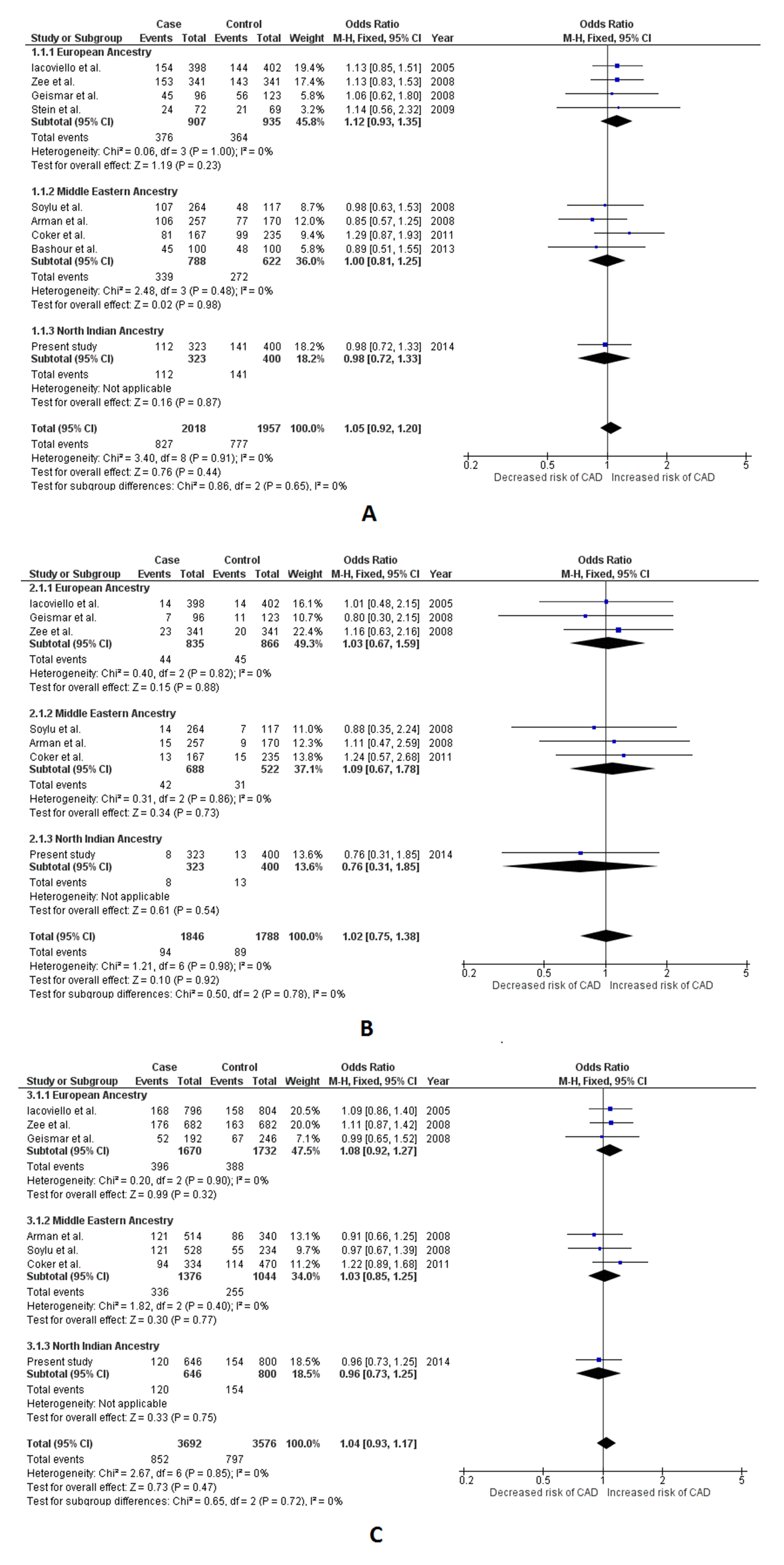

Supplement: S3 Fig — Panel A: Effect size estimation using dominant genetic model (TT+CT vs. CC); Panel B: Effect size estimation using recessive genetic model (TT vs. CT+CC); Panel C: Effect size estimation using allelic genetic model (Allele T vs. Allele C). Effect size estimates for dominant, recessive and allelic genetic models (for pooled as well as all ancestral subgroups) were obtained using fixed effects for analysis. (TIF) [file pone.0153480.s006.tif]

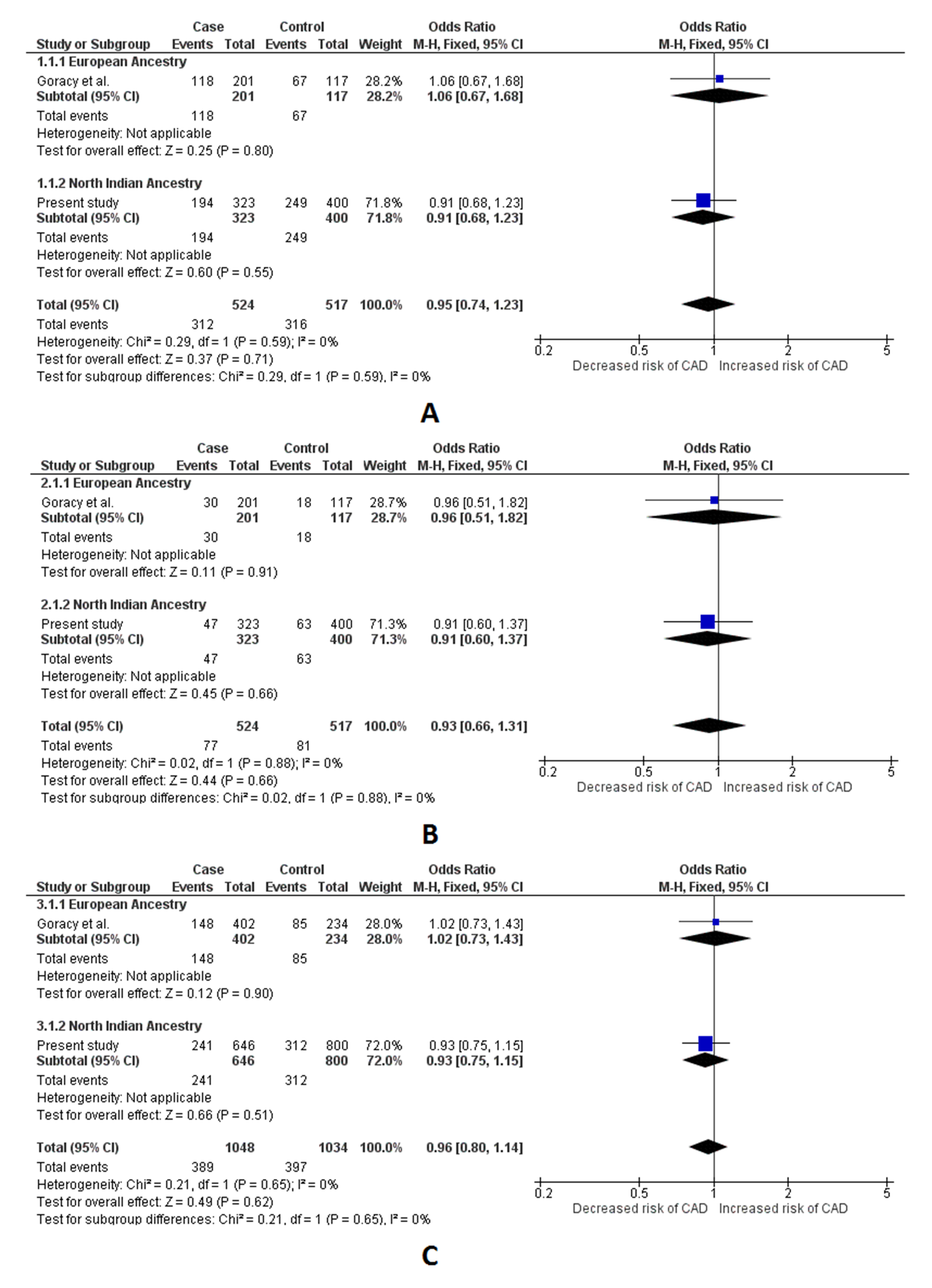

Supplement: S4 Fig — Panel A: Effect size estimation using dominant genetic model (TT+CT vs. CC); Panel B: Effect size estimation using recessive genetic model (TT vs. CT+CC); Panel C: Effect size estimation using allelic genetic model (Allele T vs. Allele C). Effect size estimates for dominant, recessive and allelic genetic models (for pooled as well as all ancestral subgroups) were obtained using fixed effects for analysis. (TIF) [file pone.0153480.s007.tif]

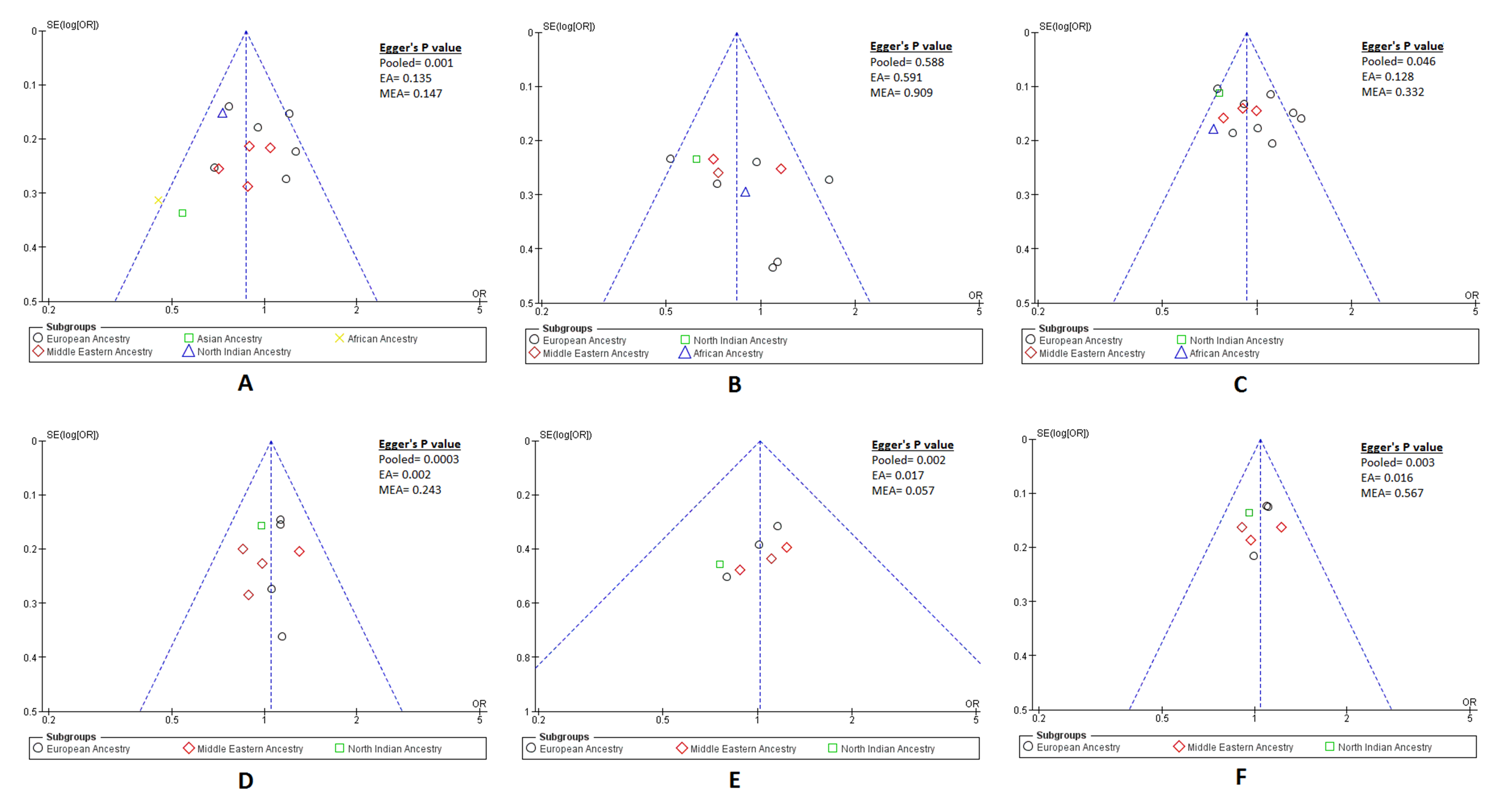

Supplement: S5 Fig — Each point in each figure represents OR of a study plotted against the standard error (SE) its OR. Different indicators of the studies belonging to each ancestral group are used in these plots. Panel A: Begg’s funnel plot with Egger’s estimates for dominant genetic model of IL1B -511 C>T polymorphism. Panel B: Begg’s funnel plot with Egger’s estimates for recessive genetic model of IL1B -511 C>T polymorphism. Panel C: Begg’s funnel plot with Egger’s estimates for allelic genetic model of IL1B -511 C>T polymorphism. Panel D: Begg’s funnel plot with Egger’s estimates for dominant genetic model of IL1B -3954 C>T polymorphism. Panel E: Begg’s funnel plot with Egger’s estimates for recessive genetic model of IL1B -3954 C>T polymorphism. Panel F: Begg’s funnel plot with Egger’s estimates for allelic genetic model of IL1B -3954 C>T polymorphism. Abbreviations- EA: European Ancestry; MEA: Middle Eastern Ancestry. (TIF) [file pone.0153480.s008.tif]

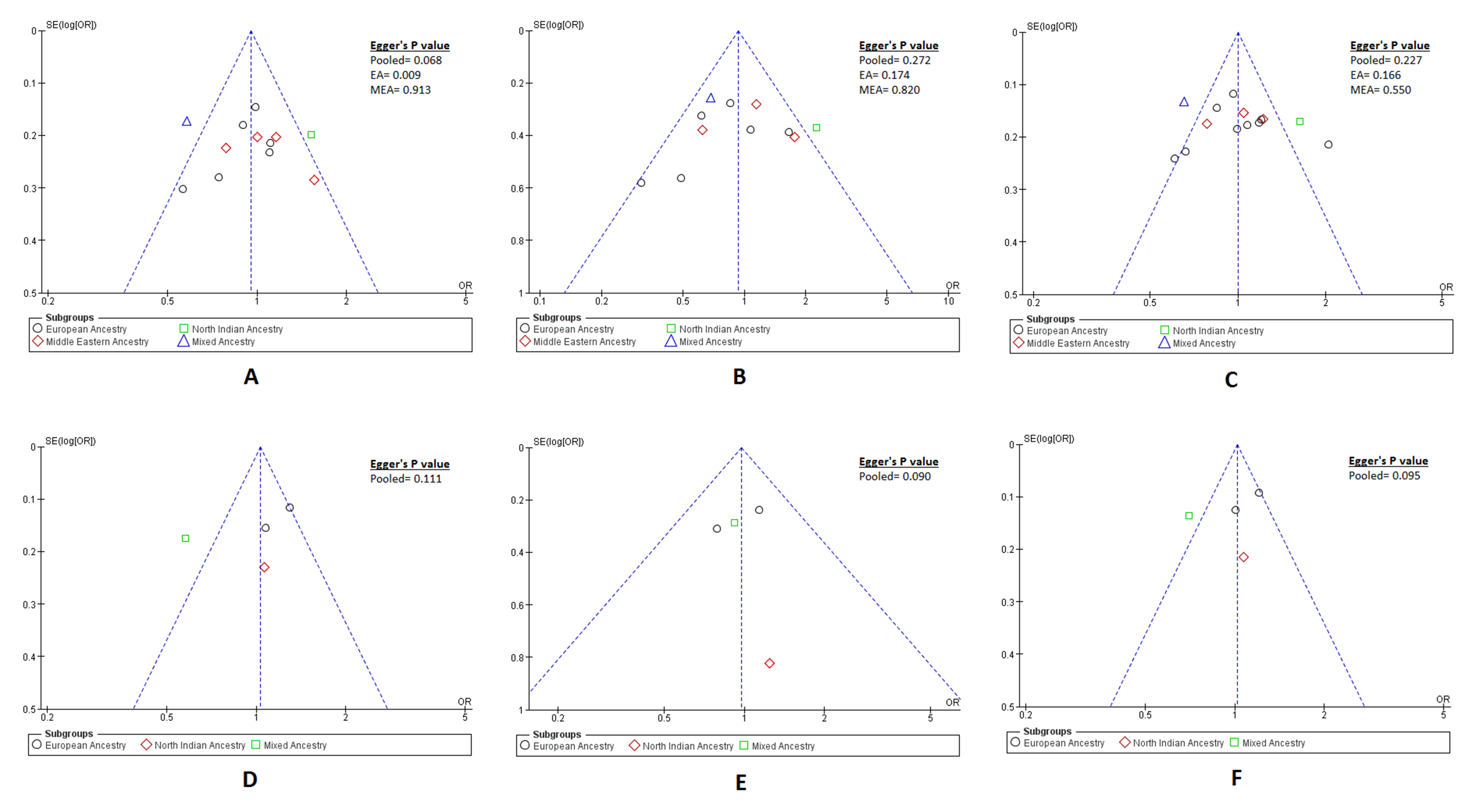

Supplement: S6 Fig — Each point in each figure represents OR of a study plotted against the standard error (SE) its OR. Different indicators of the studies belonging to each ancestral group are used in these plots. Panel A: Begg’s funnel plot with Egger’s estimates for dominant genetic model of IL1RN 86bp VNTR polymorphism. Panel B: Begg’s funnel plot with Egger’s estimates for recessive genetic model of IL1RN 86bp VNTR polymorphism. Panel C: Begg’s funnel plot with Egger’s estimates for allelic genetic model of IL1RN 86bp VNTR polymorphism. Panel D: Begg’s funnel plot with Egger’s estimates for dominant genetic model of IL1RN +8006 T>C polymorphism. Panel E: Begg’s funnel plot with Egger’s estimates for recessive genetic model of IL1RN +8006 T>C polymorphism. Panel F: Begg’s funnel plot with Egger’s estimates for allelic genetic model of IL1RN +8006 T>C polymorphism. Abbreviations- EA: European Ancestry; MEA: Middle Eastern Ancestry. (TIF) [file pone.0153480.s009.tif]
