# Supplementary material for: Interleukin-1 Gene Cluster Polymorphisms and Their Association with Coronary Artery Disease: Separate Evidences from the Largest Case-Control Study amongst North Indians and an Updated Meta-Analysis
Source: PLoS One. 2016 Apr 14;11(4):e0153480. doi: 10.1371/journal.pone.0153480 (PMC4831754; doi:10.1371/journal.pone.0153480)
Supplement: S1 File — (DOC) [file pone.0153480.s010.doc]

**List of articles excluded from the present meta-analysis.**

| **S.No.** | **Article (Reference)** | **IL-1 gene cluster SNPs studied** | **Reason for exclusion** |
| --- | --- | --- | --- |
| 1 | Olofsson et al., 2009  **(*Circ J. 2009 Aug;73(8):1531-6.*)** | *IL1RN* 86 bp VNTR | Relevant data required for inclusion in our meta-analysis, not available in the manuscript and not made available even after several request to the authors |
| 2 | Goteiner et al., 2008  **(J Periodontol. 2008 Jan;79(1):138-43.)** | *IL1A*+4845; *IL1B* -3954 | Relevant data required for inclusion in our meta-analysis, not available in the manuscript and not made available even after several request to the authors |
| 3 | Gaetano et al., 2011  **(Thromb Haemost 2011; 106: 1231–1233)** | Details not available | Relevant data required for inclusion in our meta-analysis, not available in the manuscript and not made available even after several request to the authors |
| 4 | Tsimikas et al., 2014  **(J Am Coll Cardiol. 2014 May 6;63(17):1724-34.)** | *IL1A*+4845; *IL1B* -3954;  *IL1B* -511 | Relevant data required for inclusion in our meta-analysis, not available in the manuscript and not made available even after several request to the authors |
| 5 | Bis et al., 2008  **(Atherosclerosis. 2008 May ; 198(1): 166–173.)** | *IL1B* -5887 | Relevant data required for inclusion in our meta-analysis, not available in the manuscript and not made available even after several request to the authors |
| 6 | Zhu et al., 2009  **(Sichuan Da**  **Xue Xue Bao Yi Xue Ban 40: 73–76.)** | *IL1B*+3954 | Article in Chinese |
| 7 | Zeybek et al., 2011  **(Mol Biol Rep. 2011, 38: 5453–5457)** | *IL1B*+3954 | Genotype distribution among controls not satisfying Hardy-Weinberg approximations. |
| 8 | Armingohar et al., 2014  **(Scand J Immunol. 2014 May;79(5):338-45.)** | *IL1A* -889; *IL1B* -511; *IL1B*+3954; *IL1RN* 86bp VNTR | Non relevant outcome: Study among patients of Non-coronary vascular disease. |
| 9 | Geismar et al., 2008^a^  **(J Periodontol. 2008 Dec;79(12):2322-30.)** | *IL1A*+4845 | Genotype distribution among controls not satisfying Hardy-Weinberg approximations. |
| 10 | Francis et al., 1999^b^  **(Circulation 1999, 99:861-866)** | *IL1A* -889; *IL1B*+3954 | Relevant data required for inclusion in our meta-analysis, not available in the manuscript and not made available even after several request to the authors |
| 11 | Momiyama et al., 2001^c^  **(J Am Coll Cardiol. 2001 Sep;38(3):712-7.)** | *IL1RN* 86bp VNTR | Relevant data required for inclusion in our meta-analysis, not available in the manuscript and not made available even after several request to the authors |
| 12 | Haroon et al., 2015  **(Lab Med. 2015**  **Winter;46(1):20-5. doi: 10.1309/LM1SAPZQRNQT4BO9.)** | *IL1A* -889 | Genotype distribution among controls not satisfying Hardy-Weinberg approximations. |

Footnotes: ^a^Otherwise included for data pertaining to *IL1B* -511; *IL1B*+3954 and *IL1RN* 86bp VNTR. ^b^Otherwise included for data pertaining to *IL1B* -511 and *IL1RN* 86bp VNTR. ^c^Otherwise included for data pertaining to *IL1B* -511.
