# Supplementary material for: Interleukin-1 Gene Cluster Polymorphisms and Their Association with Coronary Artery Disease: Separate Evidences from the Largest Case-Control Study amongst North Indians and an Updated Meta-Analysis
Source: PLoS One. 2016 Apr 14;11(4):e0153480. doi: 10.1371/journal.pone.0153480 (PMC4831754; doi:10.1371/journal.pone.0153480)
Supplement: S1 Table — (DOC) [file pone.0153480.s011.doc]

**S1 Table. Baseline characteristics of the study cohort.**

|  | **Cases (n= 323)** | **Controls (n= 400)** | **OR** | **95% CI** | **p value** |
| --- | --- | --- | --- | --- | --- |
| **General Characteristics** |  |  |  |  |  |
| Age (years) | 52.50 ± 12.66 (0.71) | 52.15 ± 13.14 (0.66) | - | -1.54-2.24 | 0.716 |
| Males | 276 (85.45) | 346 (86.50) | 0.92 | 0.60-1.40 | 0.746 |
| Smokers | 140 (43.34) | 127 (31.75) | 1.65 | 1.21-2.23 | 0.001^*^ |
| Non-Vegetarians | 146 (45.20) | 170 (42.50) | 1.12 | 0.83-1.50 | 0.498 |
| Alcohol consumers | 97 (30.03) | 122 (30.50) | 0.98 | 0.71-1.35 | 0.935 |
| **Other Risk factors for CAD** |  |  |  |  |  |
| Type II Diabetes | 56 (17.3) | - | - | - | - |
| Hypertension | 77 (23.8) | - | - | - | - |
| Family history of CAD | 89 (27.6) | - | - | - | - |
| **Diagnosis at Inclusion** |  |  |  |  |  |
| Myocardial Infarction (within <30 days) | 239 (74.0) | - | - | - | - |
| Unstable angina | 43 (13.3) | - | - | - | - |
| Stable angina | 31 (9.6) | - | - | - | - |
| Asymptomatic known CAD | 10 (3.1) | - | - | - | - |
| **Biochemical Characteristics** |  |  |  |  |  |
| TC (mg/dl) | 176.72 ± 52.99 (2.95) | 151.06 ± 29.81 (1.49) | - | 19.16-32.15 | <0.001^*^ |
| TG (mg/dl) | 152.46 ± 90.07 (5.01) | 139.94 ± 62.33 (3.12) | - | 0.93-24.11 | 0.034^*^ |
| HDL-c (mg/dl) | 41.97 ± 10.65 (0.59) | 38.25 ± 9.76 (0.49) | - | 2.22-5.23 | <0.001^*^ |
| LDL-c (mg/dl)^**^ | 104.98 ± 45.09 (2.52) | 82.83 ± 24.65 (1.23) | - | 14.63-25.67 | <0.001^*^ |
| VLDL-c (mg/dl)^**^ | 29.44 ± 15.25 (0.85) | 27.99 ± 12.47 (0.62) | - | -0.62-3.54 | 0.168 |
| Non HDL-c (mg/dl) | 134.75 ± 49.94 (2.78) | 112.82 ± 25.10 (1.25) | - | 15.94-27.92 | <0.001^*^ |
| TC/HDL-c ratio | 4.37 ± 1.45 (0.08) | 4.08 ± 0.79 (0.04) | - | 0.11-0.47 | 0.001^*^ |
| LDL-c/HDL-c ratio^**^ | 2.60 ± 1.22 (0.07) | 2.30 ± 0.70 (0.03) | - | 0.15-0.45 | <0.001^*^ |
| Fasting Glucose(mg/dl) | 108.08 ± 44.79 (2.49) | 78.14 ± 6.62 (0.33) | - | 24.99-34.88 | <0.001^*^ |

SD: Standard deviation; SE: Standard error (mean); OR: Odds ratio; CI: Confidence interval; TC: Total Cholesterol; TG: Triglycerides; HDL-c: High Density Lipoprotein Cholesterol; LDL-c and VLDL-c: Low and Very Low Density Lipoprotein Cholesterol.

General Characteristics among the two groups are expressed in N (%) and are compared using a Chi2 test, except for Age which is expressed in Mean ± SD (SE) and compared using a student’s T test. All Biochemical Characteristics are expressed in Mean ± SD (SE) and are compared using a student’s T test.

^*^p value of <0.05 was considered to be statistically significant; ^**^Calculated for 319 CAD patients (TG>400) vs. 400 controls.
