# Supplementary material for: Interleukin-1 Gene Cluster Polymorphisms and Their Association with Coronary Artery Disease: Separate Evidences from the Largest Case-Control Study amongst North Indians and an Updated Meta-Analysis
Source: PLoS One. 2016 Apr 14;11(4):e0153480. doi: 10.1371/journal.pone.0153480 (PMC4831754; doi:10.1371/journal.pone.0153480)
Supplement: S2 Table — (DOC) [file pone.0153480.s012.doc]

**S2 Table. Genotypic and allelic comparisons in the present case-control study.**

|  | **Cases (n= 323)** | **Controls (n= 400)** | **OR (95% CI)** | **p value^*^** |
| --- | --- | --- | --- | --- |
| ***IL1A*-889 C>T (rs1800587)** |  |  |  |  |
| TT (Mutant) | 3 (0.93%) | 4 (1.00%) | 0.93 (0.21-4.18) | 1.000 |
| CT (Heterozygous) | 54 (17.72%) | 57 (14.25%) | 1.21 (0.81-1.81) | 0.407 |
| CC (Wild type) | 266 (82.35%) | 339 (84.75%) | 0.84 (0.56-1.25) | 0.418 |
| TT vs. CC (Co-Dominant Model) | 3/266 | 4/339 | 0.96 (0.21-4.31) | 1.000 |
| CT vs. CC (Co-Dominant Model) | 54/266 | 57/339 | 1.21 (0.80-1.81) | 0.406 |
| TT+CT vs. CC (Dominant Model) | 57/266 | 61/339 | 1.19 (0.80-1.77) | 0.418 |
| TT vs. CT+CC (Recessive Model) | 3/320 | 4/396 | 0.93 (0.21-4.18) | 1.000 |
| Allele T (frequency) | 0.09 | 0.08 | 1.16 (0.80-1.67) | 0.452 |
| Allele C (frequency) | 0.91 | 0.92 | 0.86 (0.60-1.25) |  |
| ***IL1A*+4845 G>T (rs17561)** |  |  |  |  |
| TT (Mutant) | 4 (1.24%) | 7 (1.75%) | 0.70 (0.20-2.43) | 0.762 |
| GT (Heterozygous) | 60 (18.58%) | 66 (16.50%) | 1.15 (0.78-1.70) | 0.491 |
| GG (Wild type) | 259 (80.19%) | 327 (81.75%) | 0.90 (0.62-1.31) | 0.633 |
| TT vs. GG (Co-Dominant Model) | 4/259 | 7/327 | 0.72 (0.21-2.49) | 0.763 |
| GT vs. GG (Co-Dominant Model) | 60/259 | 66/327 | 1.15 (0.78-1.70) | 0.491 |
| TT+GT vs. GG (Dominant Model) | 64/259 | 73/327 | 1.11 (0.76-1.61) | 0.633 |
| TT vs. GT+GG (Recessive Model) | 4/319 | 7/393 | 0.70 (0.20-2.43) | 0.762 |
| Allele T (frequency) | 0.11 | 0.10 | 1.06 (0.75-1.49) | 0.794 |
| Allele G (frequency) | 0.89 | 0.90 | 0.94 (0.67-1.33) |  |
| ***IL1B* -511 C>T (rs16944)** |  |  |  |  |
| TT (Mutant) | 32 (9.91%) | 60 (15.00%) | 0.62 (0.39-0.98) | 0.044 |
| CT (Heterozygous) | 143 (44.27%) | 187 (46.75%) | 0.90 (0.67-1.21) | 0.548 |
| CC (Wild type) | 148 (45.82%) | 153 (38.25%) | 1.36 (1.01-1.84) | 0.041 |
| TT vs. CC (Co-Dominant Model) | 32/148 | 60/153 | 0.55 (0.34-0.89) | 0.017 |
| CT vs. CC (Co-Dominant Model) | 143/148 | 187/153 | 0.79 (0.58-1.08) | 0.151 |
| TT+CT vs. CC (Dominant Model) | 175/148 | 247/153 | 0.73 (0.54-0.99) | 0.041 |
| TT vs. CT+CC (Recessive Model) | 32/291 | 60/340 | 0.62 (0.39-0.98) | 0.044 |
| Allele T (frequency) | 0.32 | 0.38 | 0.76 (0.61-0.94) | 0.013 |
| Allele C (frequency) | 0.68 | 0.62 | 1.32 (1.06-1.64) |  |
| ***IL1B* -1903 C>T (rs1143627)** |  |  |  |  |
| TT (Mutant) | 47 (14.55%) | 63 (15.75%) | 0.91 (0.60-1.37) | 0.678 |
| CT (Heterozygous) | 147 (45.51%) | 186 (46.50%) | 0.96 (0.72-1.29) | 0.822 |
| CC (Wild type) | 129 (39.94%) | 151 (37.75%) | 1.10 (0.81-1.48) | 0.591 |
| TT vs. CC (Co-Dominant Model) | 47/129 | 63/151 | 0.87 (0.56-1.36) | 0.573 |
| CT vs. CC (Co-Dominant Model) | 147/129 | 186/151 | 0.92 (0.67-1.27) | 0.684 |
| TT+CT vs. CC (Dominant Model) | 194/129 | 249/151 | 0.91 (0.67-1.23) | 0.591 |
| TT vs. CT+CC (Recessive Model) | 47/276 | 63/337 | 0.91 (0.60-1.37) | 0.678 |
| Allele T (frequency) | 0.37 | 0.39 | 0.93 (0.75-1.15) | 0.514 |
| Allele C (frequency) | 0.63 | 0.61 | 1.07 (0.87-1.33) |  |
| ***IL1B* -3954 C>T (rs1143634)** |  |  |  |  |
| TT (Mutant) | 8 (2.48%) | 13 (3.25%) | 0.76 (0.31-1.85) | 0.658 |
| CT (Heterozygous) | 104 (32.20%) | 128 (32.00%) | 1.01 (0.74-1.38) | 1.000 |
| CC (Wild type) | 211 (65.33%) | 259 (64.75%) | 1.03 (0.75-1.39) | 0.876 |
| TT vs. CC (Co-Dominant Model) | 8/211 | 13/259 | 0.75 (0.31-1.86) | 0.656 |
| CT vs. CC (Co-Dominant Model) | 104/211 | 128/259 | 0.99 (0.73-1.37) | 1.000 |
| TT+CT vs. CC (Dominant Model) | 112/211 | 141/259 | 0.97 (0.72-1.33) | 0.876 |
| TT vs. CT+CC (Recessive Model) | 8/315 | 13/387 | 0.76 (0.31-1.85) | 0.658 |
| Allele T (frequency) | 0.19 | 0.19 | 0.96 (0.73-1.25) | 0.787 |
| Allele C (frequency) | 0.81 | 0.81 | 1.04 (0.80-1.36) |  |
| ***IL1B* -5887 C>T(rs1143633)** |  |  |  |  |
| TT (Mutant) | 28 (8.67%) | 33 (8.25%) | 1.06 (0.62-1.79) | 0.893 |
| CT (Heterozygous) | 114 (35.29%) | 139 (34.75%) | 1.01 (0.75-1.39) | 0.937 |
| CC (Wild type) | 181 (56.04%) | 228 (57.00%) | 0.96 (0.71-1.29) | 0.821 |
| TT vs. CC (Co-Dominant Model) | 28/181 | 33/228 | 1.07 (0.62-1.83) | 0.890 |
| CT vs. CC (Co-Dominant Model) | 114/181 | 139/228 | 1.03 (0.75-1.42) | 0.872 |
| TT+CT vs. CC (Dominant Model) | 142/181 | 172/228 | 1.04 (0.77-1.40) | 0.821 |
| TT vs. CT+CC (Recessive Model) | 28/295 | 33/367 | 1.06 (0.62-1.79) | 0.893 |
| Allele T (frequency) | 0.26 | 0.26 | 1.04 (0.82-1.31) | 0.809 |
| Allele C (frequency) | 0.74 | 0.74 | 0.96 (0.76-1.22) |  |
| ***IL1RN* 86bp VNTR (PMID 14563376)** | |  |  |  |
| 1/1 Genotype | 141 (43.65%) | 186 (46.5%) | 0.89 (0.66-1.20) | 0.453 |
| 1/2 Genotype | 38 (11.76%) | 43 (10.75%) | 1.11 (0.70-1.76) | 0.722 |
| 2/2 Genotype | 21 (6.50%) | 12 (3.00%) | 2.25 (1.09-4.64) | 0.031 |
| 2/4 Genotype | 7 (2.17%) | 3 (0.75%) | 2.93 (0.75-11.43) | 0.120 |
| 1/4 Genotype | 103 (31.89%) | 142 (35.50%) | 0.85 (0.62-1.16) | 0.343 |
| 4/4 Genotype | 13 (4.02%) | 14 (3.50%) | 1.16 (0.53-2.50) | 0.844 |
| Allele 1(410bp) | 423 (65.48%) | 557 (69.63%) | 0.83 (0.66-1.03) | 0.101 |
| Allele 2 (240bp) | 87 (13.47%) | 70 (8.75%) | 1.62 (1.16-2.27) | 0.005 |
| Allele 4 (325bp) | 136 (21.05%) | 173 (21.63%) | 0.97 (0.75-1.24) | 0.797 |
| ***Comparisons according to “allele 2” and “allele X” nomenclature*** | | | | |
| 2/2 Genotype | 21 (6.5%) | 12 (3%) | 2.25 (1.09-4.64) | 0.031 |
| X^#^/2 Genotype | 45 (13.93%) | 46 (11.5%) | 1.25 (0.80-1.93) | 0.367 |
| X^#^/X^#^ Genotype | 257 (79.57%) | 342 (85.5%) | 0.66 (0.45-0.97) | 0.037 |
| 2/2 + X^#^/2 vs. X^#^/X^#^ (Dominant Model) | 66/257 | 58/342 | 1.51 (1.03-2.33) | 0.037 |
| 2/2 vs. X^#^/2 + X^#^/X^#^ (Recessive Model) | 21/302 | 12/388 | 2.25 (1.09-4.64) | 0.031 |
| Allele 2 vs. Allele X^#^ | 87/559 | 70/730 | 1.62 (1.16-2.27) | 0.005 |
| ***IL1RN* +8006 T>C (rs419598)** |  |  |  |  |
| CC (Mutant) | 3 (0.93%) | 3 (0.75%) | 1.24 (0.25-6.19) | 1.000 |
| TC (Heterozygous) | 37 (11.46%) | 44 (11.00%) | 1.05 (0.66-1.66) | 0.906 |
| TT (Wild type) | 283 (87.62%) | 353 (88.25%) | 0.94 (0.60-1.48) | 0.819 |
| CC vs. TT (Co-Dominant Model) | 3/283 | 3/353 | 1.25 (0.25-6.23) | 1.000 |
| TC vs. TT (Co-Dominant Model) | 37/283 | 44/353 | 1.05 (0.66-1.67) | 0.906 |
| CC+TC vs. TT (Dominant Model) | 40/283 | 47/353 | 1.06 (0.68-1.66) | 0.819 |
| CC vs. TC+TT (Recessive Model) | 3/320 | 3/397 | 1.24 (0.25-6.19) | 1.000 |
| Allele C (frequency) | 0.07 | 0.06 | 1.07 (0.70-1.63) | 0.829 |
| Allele T (frequency) | 0.93 | 0.93 | 0.93 (0.61-1.42) |  |
| ***IL1RN* +8061 C>T (rs423904)** |  |  |  |  |
| TT (Mutant) | 25 (7.74%) | 31 (7.75%) | 0.99 (0.58-1.73) | 1.000 |
| CT (Heterozygous) | 115 (35.60%) | 137 (34.25%) | 1.06 (0.78-1.44) | 0.754 |
| CC (Wild type) | 183 (56.66%) | 232 (58.00%) | 0.95 (0.70-1.27) | 0.762 |
| TT vs. CC (Co-Dominant Model) | 25/183 | 31/232 | 1.02 (0.58-1.79) | 1.000 |
| CT vs. CC (Co-Dominant Model) | 115/183 | 137/232 | 1.06 (0.78-1.46) | 0.748 |
| TT+CT vs. CC (Dominant Model) | 140/183 | 168/232 | 1.06 (0.78-1.42) | 0.762 |
| TT vs. CT+CC (Recessive Model) | 25/298 | 31/369 | 0.99 (0.58-1.73) | 1.000 |
| Allele T (frequency) | 0.26 | 0.25 | 1.04 (0.82-1.31) | 0.807 |
| Allele C (frequency) | 0.74 | 0.75 | 0.96 (0.76-1.23) |  |
| ***IL1RN* +9589 A>T (rs454078)** |  |  |  |  |
| TT (Mutant) | 34 (10.53%) | 36 (9.00%) | 1.19 (0.73-1.95) | 0.528 |
| AT (Heterozygous) | 128 (39.63%) | 144 (36.00%) | 1.17 (0.86-1.58) | 0.354 |
| AA (Wild type) | 161 (49.85%) | 220 (55.00%) | 0.81 (0.61-1.09) | 0.178 |
| TT vs. AA (Co-Dominant Model) | 34/161 | 36/220 | 1.29 (0.77-2.15) | 0.359 |
| AT vs. AA (Co-Dominant Model) | 128/161 | 144/220 | 1.21 (0.89-1.66) | 0.231 |
| TT+AT vs. AA (Dominant Model) | 162/161 | 180/220 | 1.23 (0.92-1.65) | 0.178 |
| TT vs. AT+AA (Recessive Model) | 34/289 | 36/364 | 1.19 (0.73-1.95) | 0.528 |
| Allele T (frequency) | 0.29 | 0.27 | 1.18 (0.94-1.48) | 0.178 |
| Allele A (frequency) | 0.71 | 0.73 | 0.85 (0.67-1.07) |  |
| ***IL1RN* +111000 T>C (rs315952)** |  |  |  |  |
| CC (Mutant) | 38 (11.76%) | 50 (12.50%) | 0.93 (0.59-1.46) | 0.819 |
| CT (Heterozygous) | 128 (39.63%) | 162 (40.50%) | 0.96 (0.71-1.30) | 0.819 |
| TT (Wild type) | 157 (48.61%) | 188 (47.00%) | 1.07 (0.79-1.43) | 0.708 |
| CC vs. TT (Co-Dominant Model) | 38/157 | 50/188 | 0.91 (0.57-1.46) | 0.720 |
| CT vs. TT (Co-Dominant Model) | 128/157 | 162/188 | 0.95 (0.69-1.29) | 0.749 |
| CC+CT vs. TT (Dominant Model) | 166/157 | 212/188 | 0.94 (0.70-1.26) | 0.708 |
| CC vs. CT+TT (Recessive Model) | 38/285 | 50/350 | 0.93 (0.59-1.46) | 0.819 |
| Allele C (frequency) | 0.32 | 0.33 | 0.95 (0.76-1.18) | 0.651 |
| Allele T (frequency) | 0.68 | 0.67 | 1.05 (0.84-1.32) |  |

All calculations were performed using two tailed Fisher’s exact test with Bonferroni’s correction. Odds ratios (ORs) are reported with its 95% confidence interval.

^*^A p value of <0.0045 was considered significant (adjusted using Bonferroni’s correction). ^#^Any other allele than allele 2 of IL1RN 86bp VNTR.
