# Supplementary material for: Interleukin-1 Gene Cluster Polymorphisms and Their Association with Coronary Artery Disease: Separate Evidences from the Largest Case-Control Study amongst North Indians and an Updated Meta-Analysis
Source: PLoS One. 2016 Apr 14;11(4):e0153480. doi: 10.1371/journal.pone.0153480 (PMC4831754; doi:10.1371/journal.pone.0153480)
Supplement: S3 Table — (DOC) [file pone.0153480.s013.doc]

**S3 Table. Haplotypic distribution among cases and controls.**

| **Haplotype** | **Cases (n= 323)** | **Controls (n= 400)** | **OR (95 % CI)** | **p value**^*^ |
| --- | --- | --- | --- | --- |
| ***IL1B (-889 C>T and +4845G>T)*** | | | | |
| **Non-associated haplotypes** | | | | |
| CG | 533 (82.51%) | 670 (83.75%) | 0.91 (0.69-1.21) | 0.571 |
| CT | 53 (8.20%) | 65 (8.13%) | 1.01 (0.69-1.48) | 1.000 |
| TG | 45 (6.97%) | 50 (6.25%) | 1.12 (0.74-1.70) | 0.595 |
| 1st position: C/T = C or T allele of *IL1A* -889 C>T  2nd position: G/T = G or T allele of *IL1A* +4845G>T  *Haplotype TT had <5% of prevelance among both patient and control groups therefore was not included for calculations.* | | | | |
| **IL1B *(-511 C>T, -1903 C>T, -3954 C>T, -5887 C>T)***  **Haplotypes predisposing CAD risk** | | | | |
| CTCT | 80 (12.28%) | 36 (4.50%) | 3.00 (1.99-4.51) | <0.001^*^ |
| CCTC | 78 (12.07%) | 41 (5.13%) | 2.54 (1.71-3.77) | <0.001^*^ |
| CCCT | 56 (8.67%) | 31 (3.88%) | 2.35 (1.50-3.70) | <0.001^*^ |
| **Protective haplotypes** | |  |  |  |
| TCCT | 4 (0.62%) | 89 (11.13%) | 0.05 (0.02-0.14) | <0.001^*^ |
| CTTC | 7 (1.08%) | 69 (8.63%) | 0.12 (0.05-0.25) | <0.001^*^ |
| **Non-associated haplotypes** | |  |  |  |
| TCCC | 100 (15.48%) | 91 (11.38%) | 1.43 (1.05-1.94) | 0.023 |
| CCCC | 143 (22.14%) | 223 (27.88%) | 0.73 (0.58-0.94) | 0.013 |
| CTCC | 66 (10.22%) | 70 (8.75%) | 1.19 (0.83-1.69) | 0.365 |
| TTCC | 65 (10.06%) | 83 (10.38%) | 0.97 (0.69-1.36) | 0.862 |
| 1st position: C/T = C or T allele of *IL1B* -511 C>T  2nd position: C/T = C or T allele of *IL1B* -1903 C>T  3rd position: C/T = C or T allele of *IL1B* -3954 C>T  4th position: C/T = C or T allele of *IL1B* -5887 C>T  *Haplotypes TCTC, CCTT, TTCT, TCTT, CTTT, TTTT, TTTC had <5% of prevelance among both patient and control groups therefore were not included for calculations.* | | | | |
| ***IL1RN (+8006 T>C, +8061 C>T, +9589 A>T, +111000 T>C)*** | | | | |
| **Haplotypes predisposing CAD risk** | |  |  |  |
| TTAC | 67 (10.37%) | 23 (2.88%) | 3.91 (2.40-6.35) | <0.001^*^ |
| TCTC | 45 (6.97%) | 22 (2.75%) | 2.65 (1.57-4.46) | <0.001^*^ |
| TCTT | 101 (15.63%) | 86 (10.75%) | 1.54 (1.13-2.09) | 0.007^*^ |
| **Protective haplotypes** | |  |  |  |
| TCAC | 58 (8.98%) | 194 (24.25%) | 0.31 (0.22-0.42) | <0.001^*^ |
| **Non-associated haplotypes** | | |  |  |
| TCAT | 238 (36.84%) | 252 (31.5%) | 1.27 (1.02-1.58) | 0.034 |
| TTTT | 29 (4.49%) | 64 (8.00%) | 0.54 (0.34-0.85) | 0.007 |
| TTAT | 48 (7.43%) | 91 (11.38%) | 0.62 (0.43-0.90) | 0.012 |
| 1st position: T/C = T or C allele of *IL1RN* +8006 T>C  2nd position: C/T = C or T allele of *IL1RN* +8061 C>T  3rd position: A/T = A or T allele of *IL1RN* +9589 A>T  4th position: T/C = T or C allele of *IL1RN* +111000 T>C  *Haplotypes CCAC, TTTC, CCAT, CTAC, CCTT, CTAT, CCTC had <5% of prevelance among both patient and control groups therefore were not included for calculations.* | | | | |

Haplotypes were generated from multi-locus diploid data based on a Gibbs sampling strategy (Arlequin v3.5). The difference in haplotype frequencies between the case and control groups for the prevalent haplotypes (with prevalence more than 5% in either cases or controls) were analyzed for statistical significance using Fisher’s exact test with Bonferroni’s correction. Odds ratios (ORs) are reported with its 95% confidence interval.

^*^p<0.045 was considered as statistically significant (adjusted as per Bonferroni’s correction).
