# Supplementary material for: Interleukin-1 Gene Cluster Polymorphisms and Their Association with Coronary Artery Disease: Separate Evidences from the Largest Case-Control Study amongst North Indians and an Updated Meta-Analysis
Source: PLoS One. 2016 Apr 14;11(4):e0153480. doi: 10.1371/journal.pone.0153480 (PMC4831754; doi:10.1371/journal.pone.0153480)
Supplement: S4 Table — (DOC) [file pone.0153480.s014.doc]

**S4 Table. Meta-analysis: Basic characteristics of the studied groups.**

|  | **Heterogeneity (*I^2^;* P_Q_)** | | | **Number of**  **Studies in each model** | **Cases (n) / Controls (n)** | **Total sample**  **assessed** |
| --- | --- | --- | --- | --- | --- | --- |
|  | **Dominant genetic model (D)^a^** | **Recessive genetic model (R)^b^** | **Allelic genetic model (A)^c^** |  |  |  |
| ***IL1A* polymorphisms** |  |  |  |  |  |  |
| **-889 C>T (rs1800587)** |  |  |  |  |  |  |
| Pooled | 0%; 0.98 | 0%; 0.72 | 0%; 0.88 | D= 3; R & A= 2 | D= 605/701; R & A= 533/632 | 1,306  (3 studies) |
| European Ancestry | - | - | - | D= 1; R= 0; A= 0 | D= 72/69; R & A= 0/0 |  |
| North Indian Ancestry | 0%; 0.84 | 0%, 0.72 | 0%, 0.88 | D, R & A= 2 | D, R & A= 533/632 |  |
| **+4845 G>T (rs17561)** |  |  |  |  |  |  |
| Pooled | 85%; 0.001 | 0%; 0.52 | 0%; 0.83 | D= 3; R &A= 2 | D= 764/841; R & A= 664/741 | 1,605  (3 studies) |
| European Ancestry | - | - | - | D, R & A= 1 | D, R & A= 341/341 |  |
| Middle Eastern Ancestry | - | - | - | D= 1, R &A= 0 | D= 100/100; R & A= 0/0 |  |
| North Indian Ancestry | - | - | - | D, R & A= 1 | D, R & A= 323/400 |  |
| ***IL1B* polymorphisms** |  |  |  |  |  |  |
| **-511 C>T (rs16944)** |  |  |  |  |  |  |
| Pooled | 38%; 0.08 | 39%; 0.09 | 54%; 0.01 | D= 13; R= 11; A= 13 (total 15 different studies) | D= 3,703/2,821; R= 2,742/2,385; A= 3,415/2,617 | 7,429  (15 studies) |
| European Ancestry | 41%; 0.13 | 58%; 0.04 | 60%; 0.02 | D, R= 6; A= 8 | D, R= 1,593/1,348; A= 2,266/1,580 |  |
| Middle Eastern Ancestry | 0%; 0.73 | 21%; 0.28 | 0%; 0.52 | D= 4; R & A= 3 | D= 788/622, R & A= 688/522 |  |
| Asian Ancestry | - | - | - | D= 1; R & A= 0; | D= 188/104, R & A= 0/0 |  |
| North Indian Ancestry | - | - | - | D, R & A= 1 | D, R & A= 323/400 |  |
| African Ancestry | - | - | - | D, R & A= 1 | D, R & A= 138/115 |  |
| **-1903 C>T (rs1143627)** |  |  |  |  |  |  |
| Pooled | 0%; 0.59 | 0%; 0.88 | 0%; 0.65 | D, R & A= 2 | D, R & A= 524/517 | 1,041  (2 studies) |
| European Ancestry | - | - | - | D, R & A= 1 | D, R & A= 201/117 |  |
| North Indian Ancestry | - | - | - | D, R & A= 1 | D, R & A= 323/400 |  |
| **-3954 C>T (rs1143634)** |  |  |  |  |  |  |
| Pooled | 0%; 0.91 | 0%; 0.98 | 0%; 0.85 | D= 9; R & A= 7 | D= 2,018/1,957; R & A= 1,846/1,788 | 3,975  (9 studies) |
| European Ancestry | 0%; 1.00 | 0%; 0.82 | 0%; 0.90 | D= 4; R & A= 3 | D= 907/935; R & A= 835/866 |  |
| Middle Eastern Ancestry | 0%; 0.48 | 0%; 0.86 | 0%; 0.40 | D= 4; R & A= 3 | D= 788/622; R & A= 688/522 |  |
| North Indian Ancestry | - | - | - | D, R & A= 1 | D, R & A= 323/400 |  |
| **-5887 C>T (rs1143633)** |  |  |  |  |  |  |
| Pooled | 0%; 0.46 | 0%; 0.55 | 0%; 0.40 | D, R & A= 2 | D, R & A= 664/741 | 1,405  (2 studies) |
| European Ancestry | - | - | - | D, R & A= 1 | D, R & A= 341/341 |  |
| North Indian Ancestry | - | - | - | D, R & A= 1 | D, R & A= 323/400 |  |
| ***IL1RN* polymorphisms** |  |  |  |  |  |  |
| **86bp VNTR (PMID 14563376)** |  |  |  |  |  |  |
| Pooled | 52%; 0.02 | 50%; 0.03 | 70%; <0.0001 | D= 12; R= 11; A= 14 (total 15 different studies) | D= 2,688/2,398; R= 2,588/ 2,298; A= 3,419/2,683 | 6,302  (15 studies) |
| European Ancestry | 0%; 0.44 | 38%; 0.15 | 64%; 0.004 | D & R= 6; A= 9 | D & R= 1,277/1,128; A= 2,108/1,513 |  |
| Middle Eastern Ancestry | 23%; 0.28 | 45%; 0.16 | 43%; 0.17 | D= 4; R & A= 3; | D= 788/622; R & A= 688/522 |  |
| North Indian Ancestry | - | - | - | D, R & A= 1 | D, R & A= 323/400 |  |
| Mixed Ancestry | - | - | - | D, R & A= 1 | D, R & A= 300/248 |  |
| **+8006 T>C (rs419598)** |  |  |  |  |  |  |
| Pooled | 80%; 0.002 | 0%; 0.80 | 72%; 0.01 | D, R & A= 4 | D, R & A= 1,512/1,635 | 3,147  (4 studies) |
| European Ancestry | 0%; 0.33 | 0%; 0.35 | 24%; 0.25 | D, R & A= 2 | D, R & A= 900/987 |  |
| North Indian Ancestry | - | - | - | D, R & A= 1 | D, R & A= 323/400 |  |
| Mixed Ancestry | - | - | - | D, R & A= 1 | D, R & A= 289/248 |  |

Heterogeneity was tested among groups of studies using Higgin’s *I^2^* statistics and Cochran’s Q statistics (P_Q_). Group showing P_Q_<0.1 were considered heterogeneous, others were considered homogenous.

^a^Dominant genetic model: *TT+CT* vs. *CC* for A-889 C>T; *TT+GT* vs. *GG* for A+4845 G>T; *TT+CT* vs. *CC* for B-511 C>T; *TT+CT* vs. *CC* for B-1903 C>T; *TT+CT* vs. *CC* for B-3954 C>T; *TT+CT* vs. *CC* for B-5887 C>T; *2/2+X/2* vs. *X/X* for RN 86bp VNTR (*X*= Any other allele than *allele 2*); *CC+CT* vs. *TT* for RN+8006 T>C.

^b^Recessive genetic model: *TT* vs. *CT+CC* for A-889 C>T; *TT* vs. *CT+GG* for A+4845 G>T; *TT* vs. *CT+CC* for B-511 C>T; *TT* vs. *CT+CC* for B-1903 C>T; *TT* vs. *CT+CC* for B-3954 C>T; *TT* vs. *CT+CC* for B-5887 C>T; *2/2* vs. *X/2+X/X* for RN 86bp VNTR; *CC* vs. *CT+TT* for RN+8006 T>C.

^c^Allelic genetic model: *Allele T* vs. *Allele C* for A-889 C>T; *Allele T* vs. *Allele G* for A+4845 G>T; *Allele T* vs. *Allele C* for B-511 C>T; *Allele T* vs. *Allele C* for B-1903 C>T; *Allele T* vs. *Allele C* for B-3954 C>T; *Allele T* vs. *Allele C* for B-5887 C>T; *Allele 2* vs. *Allele X* for RN 86bp VNTR; *Allele C* vs. *Allele T* for RN+8006 T>C.
